# A Bifunctional Cytochrome P450 Enzyme Catalyzes Hydroxylation and Aryl‐Aryl Ether Formation in the Biosynthesis of Emestrin

**DOI:** 10.1002/chem.202503453

**Published:** 2025-12-26

**Authors:** Yu‐Chuan Chen, Jing‐Jing Wu, Ming‐Hua Chen, Shu‐Ming Li

**Affiliations:** ^1^ Fachbereich Pharmazie, Institut für Pharmazeutische Biologie und Biotechnologie Philipps‐Universität Marburg Marburg Germany; ^2^ Institute of Medicinal Biotechnology Chinese Academy of Medical Sciences and Peking Union Medical College Beijing P. R. China

**Keywords:** aryl‐aryl ether formation, bifunctional enzyme, cytochrome P450, emestrin, natural product biosynthesis

## Abstract

Emestrins, a subgroup of epipolythiodioxopiperazines, are originated from *cyclo*‐l‐Phe‐l‐Phe and feature a dihydrooxepine ring. They contain typically a 15‐membered lactone ring with an aryl‐aryl ether linkage. Despite considerable progress in elucidating epipolythiodioxopiperazine biosynthesis, the enzymatic mechanism for the ether bond formation in emestrins remains uncharacterized. We identified a putative gene cluster (*eme*) in the fungus *Emericella quadrilineata* with three unknown P450 enzymes, EmeE, EmeR, and EmeO. Gene deletion, feeding experiments, and *in vitro* assays proved that EmeE and EmeR install regioselective and stereospecific hydroxyl groups at the *ß*‐positions of the diketopiperazine ring. EmeO acts as a bifunctional enzyme for the construction of the lactone ring *via* an aryl‐aryl ether bond formation and simultaneous hydroxylation between phenolic and nonphenolic aromatic rings. To the best of our knowledge, such enzymatic reactions have not been reported prior to this study.

## Graphical abstract

Conversion of emestrin J (**5**) to emestrin (**1**) by three P450 enzymes from the cluster (*eme*) in *Emericella quadrilineata*. EmeO acts as a bifunctional enzyme for the construction of the 15‐membered lactone ring via an aryl‐aryl ether bond formation and simultaneous hydroxylation between phenolic and nonphenolic aromatic rings, while EmeE and EmeR install regioselective and stereospecific hydroxyl groups at the *ß*‐positions of the diketopiperazine ring.

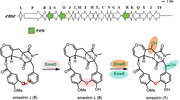

## 1 Introduction

**FIGURE 1:**
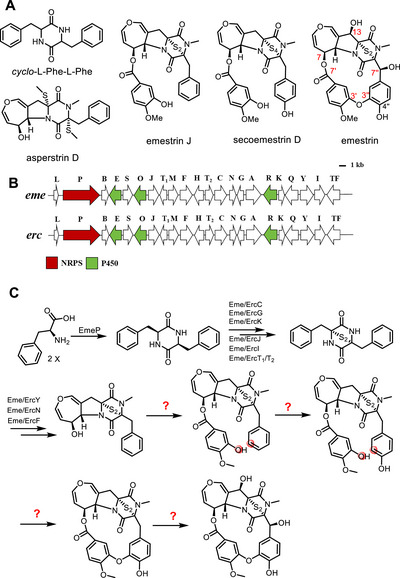
Structures of emestrin and possible precursors (A), illustration of *eme* (*E. quadrilineata* ACCC31557) and *erc* clusters (*A. nidulans* 1454) (B), and possible biosynthetic pathway of emestrin (C). Genes of interest in this work are highlighted in color.

Epipolythiodioxopiperazines (ETPs) are an important class of microbial natural products, typically derived from a cyclodipeptide with at least one aromatic amino acid and characterized by a sulfide bridge [1, 2]. Due to their structural diversity and significant biological activities such as antifungal [3] and cytotoxic [4] activities, ETPs have attracted extensive research interest [1, 2]. Emestrin (Figure 1A), an ETP derivative with a distinctive 15‐membered lactone and a dihydrooxepine ring, was first isolated from *Emericella striata* [5], followed by the identification of a number of analogues [1, 6, 7, 8] and putative biosynthetic precursors such as *cyclo*‐l‐Phe‐l‐Phe (cFF) [9], asperstrin D [8], emestrin J [3], and secoemestrin [10] (Figure 1A). Emestrin J and secoemestrin D differ from emestrin by lacking the aryl‐aryl ether bond (AAEB) and the two hydroxyl groups at the *ß*‐positions of the diketopiperazine ring. Emestrin J was equipped with a disulfide‐ instead of a tetrasulfide‐bridge in secoemestrin D.

Intensive studies on the biosynthesis of ETPs such as gliotoxin [11, 12, 13, 14, 15], sirodesmin PL [16], aspirochlorine [17, 18, 19], and acetylaranotin [9] mainly focused on the formation of cyclodipeptides, the sulfide‐bridges, the pyrrolidine ring, and the dihydrooxepine moiety. Like aspirochlorine and acetylaranotin, emestrin is also derived from cFF, but with an additional structure element, that is an isovanillyl moiety linked to the two original phenylalanyl residues through an AAEB and an ester bond.

Until now, enzyme‐catalyzed AAEB formation has been only reported in limited cases. The P450 enzyme Bmp7 from the bacterium *Pseudoalteromonas luteoviolacea* catalyzes dimerization of 2,4‐dibromophenol *via* an ether bond by attacking the *ortho*‐position of one hydroxyl group with another one (Figure S1A) [20]. Similar mechanisms were also described for other P450 enzymes in the AAEB formation, like Cih33, DmlH, and EpcH in the biosynthesis of cihanmycin C [21] (Figure S1B) as well as OxyA, OxyB, and OxyE in that of teicoplanin [22] (Figure S1C). The Cu oxidase PtaE from *Pestalotiopsis fici* can mediate the conversion of a benzophenone to an aryl‐aryl ether product (Figure S1D) [23]. Furthermore, AAEB can also be formed by nonreducing polyketide synthase after assembling of the aromatic core structure (Figure S1E) [24].

In a previous study, emestrin was isolated from *Aspergillus nidulans* 1454, and a putative *erc* cluster was proposed to consist of 22 genes (Figure 1B), being responsible for the biosynthesis of emestrin and derivatives thereof [6]. As given in Figure 1C, the enzymes for the formation of cFF, the disulfide‐bridge, the pyrrolidine ring, and the dihydrooxepine moiety can be assigned by sequence analysis and comparison. However, the enzymes for the formation and attachment of the isovanillyl moiety, the hydroxylations at the benzene ring and the *ß*‐positions of the diketopiperazine ring, as well as the AAEB formation, remain unknown. It was proposed that the aromatic ring of the original phenylalanyl residue would be first hydroxylated, followed by AAEB formation in analogy to Bmp7 [20], Cih33/DmlH/EpcH [21], OxyA, OxyB, and OxyE [22] (Figures 1C and S1) [6]. However, no candidate with high sequence identities to Bmp7 or Cih33 was identified in the putative *erc* cluster.

## 2 Results and Discussion

**FIGURE 2:**
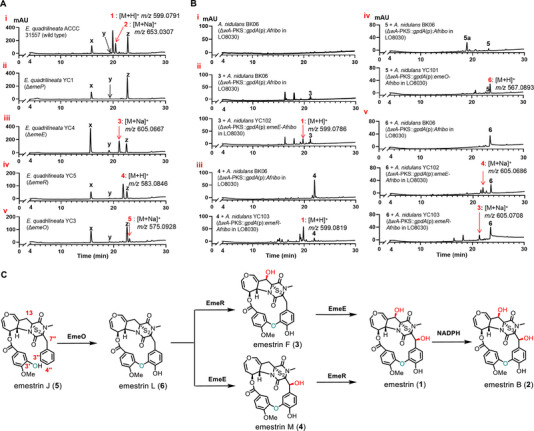
LC‐MS profiles of the metabolites from *E. quadrilineata* (A) and *A. nidulans* strains (B), as well as conversion of emestrin J (**5**) to emestrin (**1**) by the three P450 enzymes (C) The chromatograms are illustrated for UV absorptions at 254 nm. The metabolites were also detected with their [M+H]^+^ or [M+Na]^+^ ions. The *A. nidulans* transformants had been fed with the respective substrates before. Three additional peaks, **x**, **y**, and **z**, were detected in panel Ai‐v. Peak **x** at 15.8 min is a red pigment formed during sporulation. Due to very low quantity, the structure of peak **y** at 19 min could not be determined. Peak **z** at 22.8 min was isolated and determined to be sterigmatocystin by ^1^H NMR and ^13^C NMR analyses (data not shown).

To identify enzyme(s) responsible for the AAEB formation, we used *E. quadrilineata* ACCC 31557, a strain with a high emestrin yield, for our genetic study. As shown in (Figure 2Ai), emestrin (**1**) and its trisulfide congener emestrin B [25] (**2**) were clearly detected as main products in rice culture after 10 days. Due to the similar NMR data with emestrin, X‐ray diffraction analysis was used to confirm the structure of emestrin B (Table S1, CCDC 2488010) [26]. Conversion of **1** to **2** was detected in the presence of NADPH, analogues with less or more sulfur atoms were also detected after 4 hours of incubation (Figure S2). The extra sulfur atoms in emestrin B is likely from another molecule of emestrin, similar as reported for chetomin A, previously [27]. Mining of the ACCC 31557 genome revealed the presence of a homologous *eme* cluster with the same relative position and orientation of the putative genes as well as very high sequence identifies of at least 99.2% to those of the *erc* cluster (Table S2, Figure 1B).

To investigate the gene functions in the *eme* cluster, we carried out gene deletion experiments in ACCC 31557 by split‐marker strategy using hygromycin as a selection marker (Tables S3−S5, Figure S3). Metabolite changes were monitored on LC‐MS after cultivation in rice medium at 25°C for 10 days. The newly accumulated metabolites were isolated, and their structures were elucidated by interpretation of their spectroscopic data, including UV, MS, NMR, and ECD (electronic circular dichroism).

Knockout of the nonribosomal peptide synthetase gene *emeP* in ACCC 31557 resulted in complete abolishment of **1** and **2** production in the Δ*emeP* mutant YC1 (Figure 2Aii), confirming the *eme* cluster for emestrin biosynthesis. To find the candidates for the AAEB formation, three unknown cytochrome P450 enzymes raised our attention. EmeE, EmeO, and EmeR in the *eme* cluster share sequence identities of more than 99.8% with their corresponding orthologues ErcE, ErcO, and ErcR in the *erc* cluster. All three enzyme pairs show very low sequence identities (< 35%) to known proteins (Table S2). Phylogenetic analysis revealed that EmeE, EmeO, and EmeR are distributed in different clades, but not in those of AAEB‐forming enzymes like Bmp7 [20], Cih33, DmlH, EpcH [21], OxyA, OxyB, and OxyE [22] (Figure S4). EmeR is located near several hydroxylases and could be expected to catalyze one hydroxylation reaction. The function of EmeE and EmeO cannot be predicted by sequence and phylogenetic analyses. Interestingly, EmeO is located in the clade of several epoxidation enzymes such as TdaG [28], Tri4 [29], and AstB [30].

Deletion of *emeE* and *emeR* in ACCC 31557 resulted in the abolishment of both **1** and **2**. Instead, one new dominant peak each, that is **3** in YC4 (Δ*emeE*) and **4** in YC5 (Δ*emeR*), was detected, which was identified as a 7´´‐deoxy [31] and 13‐deoxy analogue of emestrin (**1**), respectively (Figure 2Aiii, iv). Compound **4**, with the same molecular formula C_27_H_22_N_2_O_9_S_2_ as **3** based on its [M+H]^+^ ion at *m/z* 583.0846, has not been described before. In the ^1^H NMR spectrum of **4** (Table S9, Figure S17), the signals of the oxymethine at C‐13 (H‐13 and OH‐13) of **1** were replaced by two doubles at *δ*_H_ 3.19 and 3.88 ppm with a coupling constant of 18.0 Hz. In addition, the chemical shift of C‐13 in the ^13^C NMR spectrum (Figure S18) was upfield shifted by Δ*δ_C_* −40.3 and −45.3 ppm, in comparison with those of **1** [6] and **2**, respectively. DEPT‐135° spectrum (Figure S19) also confirmed the methylene character of C‐13. The HMBC correlations (Figure S22) from H_2_‐13 to C‐11, C‐12, and C‐14 further supported **4** as a 13‐deoxy congener of emestrin (**1**), termed emestrin M in this study.

To confirm their function, *emeE* and *emeR* were heterologous expressed in *A. nidulans* LO8030 (Tables S3−S5, Figure S5), a modified strain from LO1362, which is derived from *A. nidulans* FGSC A4 [32]. This strain has been widely used for heterologous expression of fungal biosynthetic gene clusters due to a clean secondary metabolite background, high productivity of diverse metabolites, ease of genetic manipulation, and efficient homologous recombination [33]. Furthermore, no *erc*/*eme* homologous cluster was identified in the genome sequence of *A. nidulans* FGSC A4 strain (GCA_000011425.1) [34, 35]. The *A. nidulans* transformants were subsequently cultivated in LMM medium (see Supporting Information for details) for feeding with the respective substrates. Addition of **3** to the *emeE* overexpression strain and **4** to *emeR* overexpression strain led clearly to the formation of **1**, proving that both **3** and **4** act as direct precursors of **1**. No conversion of **3** and **4** was observed in the control strain BK06 (Figure 2Bi–iii).[36, 37]

The third uncharacterized P450 enzyme EmeO shares 33.4% identity with the bifunctional enzyme Tri4 (epoxidation and hydroxylation) in the biosynthesis of trichothecenes.[29, 38] Disruption of *emeO* in ACCC 31557 also resulted in abolishment of **1** production in the Δ*emeO* mutant YC3, while a new peak **5** appeared instead (Figure 2Av). Structural elucidation revealed the absence of the AAEB in **5**. More interestingly, in contrast to those of **1−4**, the benzene ring of one original phenylalanyl residue remains monosubstituted and carries no hydroxyl group. These results proved that EmeO was required for the ether bond formation between a phenolic hydroxyl group and a nonphenolic benzene ring, differing distinctively from other enzymes like Bmp7 [20] and Cih33/DmlH/EpcH [21] involved in the AAEB between two phenolic ring systems (Figure S1).

Inspection of the structure of **5** revealed that the hydroxyl groups at C‐13 in **1−3** and C‐7´´ in **1**, **2**, and **4** are also absent. It is likely that **5** was converted by EmeO to a product, which can be metabolized by both EmeE and EmeR. To prove the product of EmeO, **5** was fed to the *emeO* overexpression strain, resulting in almost complete conversion to **6** (Figure 2Biv). **6** was identified as a new emestrin analogue with a molecular formula of C_27_H_22_N_2_O_8_S_2_, two oxygen atoms less than **1**. Similar to that of **5**, ^1^H NMR data of **6** (Table S11, Figure S24) also suggested the absence of the two oxymethine signals of **1** at C‐13 and C‐7′′. Instead, resonances of two coupling methylene groups with large coupling constants appear at 3.22 and 3.92 (*J* = 17.8 Hz) as well as 3.58 and 3.85 ppm (*J* = 13.6 Hz). This conclusion was supported by the ^13^C NMR and DEPT‐135° spectra (Figures S25 and S26). In comparison to those of **1** [6], the chemical shifts of C‐13 and C‐7′′ in **6** are upfield shifted by Δ*δ*_C_ −34.3 and −37.6 ppm, respectively. The HMBC correlations (Figure S29) from H_2_‐13 to C‐12 and C‐14 as well as H_2_‐7′′ to C‐3, C‐4, C‐2′′, and C‐6′′ further confirm the presence of two CH_2_ groups at C‐13 and C‐7′′ in **6**. Compared to **5**, a 15‐membered lactone ring is formed in **6** *via* aryl‐aryl ether bond between the original phenolic and nonphenolic benzene ring. Meanwhile, a hydroxyl group is attached to C‐4′′.We noticed that, in the control strain *A. nidulans* BK06 [39], a distinct peak **5a** at 18.7 min with a [M+Na]^+^ ion at *m/z* 511.1502 (calcd for C_27_H_24_N_2_O_7_Na^+^: 511.1476) was detected after feeding with **5**. Unfortunately, the structure of **5a** could not be determined, due to the low quantity. However, cultivation of *emeO* overexpression strain with **5a** did not lead to detectable conversion to **6**, neither with UV, nor with ion detection (Figure S6). This excludes **5a** as an intermediate of **5** to **6** and suggested that EmeO function as a bifunctional enzyme for aryl‐aryl ether bond formation and hydroxylation.

**FIGURE 3:**
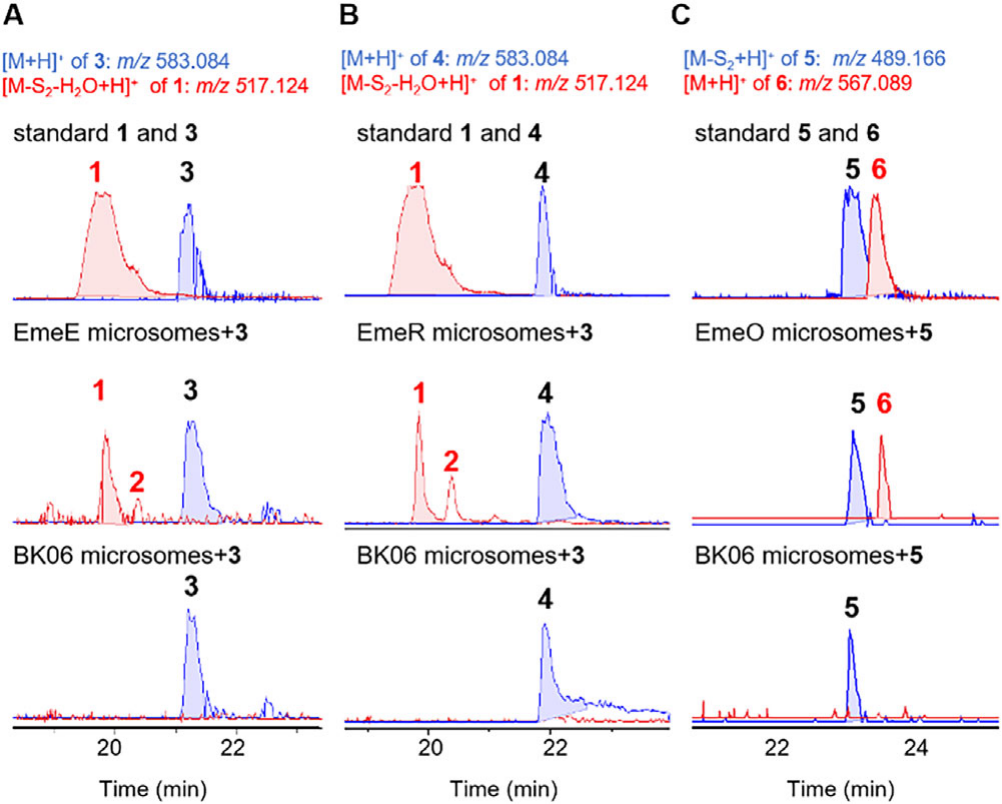
LC‐MS profiles of *in vitro* assays with microsomes of EmeE (A), EmeR (B), and EmeO (C) toward substrates **3**, **4**, and **5**, respectively. Detection was carried out by their characteristic ions with a tolerance range of ±0.005.

For *in vitro* investigation, microsomal fractions containing EmeO, EmeE, and EmeR were prepared from respective *A. nidulans* strains, according to a published protocol [40], and used for incubation at 25°C for 2 hours. In comparison to those with microsomes from the control strain BK06, LC‐MS analysis showed clearly conversion of **3** and **4** to **1** by EmeE and EmeR. In addition, **2** was also detected, being consistent with the conversion of **1** to **2** in the presence of NADPH mentioned above (Figure S2). **6** was detected as the mere product in the incubation mixture of **5** with EmeO (Figures 2C and 3).

Feeding of **6** to *emeE* and *emeR* overexpression strain led to its conversion to **4** and **3**, respectively (Figure 2Bv). This proved that **6** can serve as a precursor for both EmeE and EmeR, which catalyze regioselective and stereospecific hydroxylations at C‐7´´ and C‐13, respectively. Obviously, the two hydroxylases EmeR and EmeE show relatively broad substrate flexibility, accepting not only **6**, but also **3** or **4** as substrates (Figure 2Bii, iii). Therefore, their reaction order cannot be determined in this study. Nevertheless, conversion of **5** to **1** and then to **2** can be summarized in Figure 2C.

**SCHEME 1:**
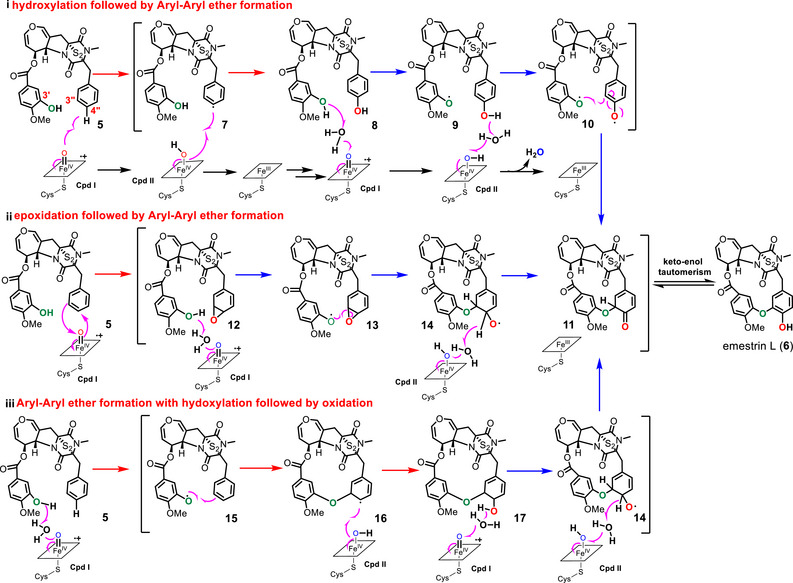
The proposed mechanisms for EmeO. The arrows for the first and second steps of EmeO are highlighted in red and blue, respectively.

Our data proved that EmeO acts as a bifunctional enzyme for the AAEB formation between a phenolic and a nonphenolic benzene ring as well as hydroxylation at the *ortho*‐position. It can be therefore proposed that the monosubstituted benzene is first hydroxylated and then attacked by the hydroxyl group from the second ring, as described for reported P450s (Scheme 1i) [21]. However, phylogenetic analysis revealed that most of the known P450s for AAEB are located in one clade, and EmeO is a member of the clade containing several epoxidation and hydroxylation [28, 29, 30]. It can be therefore also possible that an epoxide intermediate is involved in the reaction and the AAEB is formed by attack of the epoxide with the hydroxy group of another ring. The *ortho*‐hydroxyl group is the result of the epoxide ring opening and subsequent oxidation (Scheme 1ii).

The third plausible mechanism is AAEB formation via direct attack of the monosubstituted benzene ring by the C‐3′‐OH, followed by hydroxylation and oxidation (Scheme 1Biii). In all three mechanisms, the enol‐form **11** of emestrin L (**6**) is formed.

## 3 Conclusion

In summary, we identified in this study a gene cluster for the biosynthesis of emestrin and congeners in *E. quadrilineata* ACCC 31557. Gene deletion, feeding experiments, and *in vitro* assays demonstrated that the three uncharacterized P450 enzymes EmeO, EmeE, and EmeR are responsible for the conversion of emestrin J (**5**) to emestrin (**1**). EmeO acts as a bifunctional enzyme for the construction of the 15‐membered lactone ring *via* an AAEB formation and simultaneous hydroxylation. EmeE and EmeR catalyze afterward regioselective and stereospecific hydroxylations at the *ß*‐positions of the diketopiperazine ring. To the best of our knowledge, EmeO is the first reported enzyme catalyzing an AAEB formation between phenolic and nonphenolic benzene rings.

## Conflicts of Interest

The authors declare no conflict of interest.

## Supporting information



The supporting information includes details of experimental procedures, supplementary Tables, and Figures. The authors have cited six additional references within the Supporting Information [41, 42, 43, 44, 45, 46].
**Supporting File**: chem70630‐sup‐0001‐SuppMat.pdf.
