# Supplementary material for: A Bifunctional Cytochrome P450 Enzyme Catalyzes Hydroxylation and Aryl‐Aryl Ether Formation in the Biosynthesis of Emestrin
Source: Chemistry. 2025 Dec 26;32(7):e03453. doi: 10.1002/chem.202503453 (PMC12910431; doi:10.1002/chem.202503453)
Supplement: Supplementary file 1 — The supporting information includes details of experimental procedures, supplementary Tables, and Figures. The authors have cited six additional references within the Supporting Information [41, 42, 43, 44, 45, 46]. Supporting File: chem70630‐sup‐0001‐SuppMat.pdf. [file CHEM-32-e03453-s001.pdf]

Supporting Information

A Bifunctional Cytochrome P450 Enzyme Catalyzes Hydroxylation and Aryl-Aryl Ether Formation in the Biosynthesis of Emestrin

Yu-Chuan Chen,[a] Jing-Jing Wu,[a] Ming-Hua Chen,*[b] and Shu-Ming Li*[a]

[a] Y.-C. Chen, J.-J. Wu, Prof. S.-M. Li
Philipps-Universität Marburg, Fachbereich Pharmazie
Institut für Pharmazeutische Biologie und Biotechnologie
Robert-Koch-Straße 4, 35037 Marburg, Germany
E-mail: shuming.li@staff.uni-marburg.de

[b] Prof. M.-H. Chen
Chinese Academy of Medical Sciences and Peking Union Medical College
Institute of Medicinal Biotechnology
Beijing 100050, P. R. China
E-mail: chenminghua@imb.pumc.edu.cn

List of Contents

Experimental Procedures........ 4
General experimental procedures 4
Fungal strain and genome sequencing 4
Extraction of genomic DNA for genetic manipulation 5
Polymerase chain reaction (PCR) 5
Construction of plasmids 5
Fungal transformation 6
Fermentation, extraction and isolation. 7
Heterologous expression and feeding experiments 8
Microsome preparation and *in vitro* assays of **3**–**5** for EmeE, EmeR, and EmeO 8
Conversion of **1** to **2** in the presence of NADPH 8
X-ray diffraction analysis 9
Structural elucidation 9
Physiochemical properties 11
Supplementary Tables........ 13
Table S1. Crystal data and structure of emestrin B (**2**) 13
Table S2. Similarities and putative functions of proteins encoded by the *eme* cluster in *A. quadrilineata* ACCC 31557 and the *erc* cluster in *Emericella. nidulans* 1454. 14
Table S3. Fungal strains created and used in this study. 15
Table S4. Primers used in this study. 16
Table S5. Plasmids constructed in this study. 17
Table S6. NMR data of emestrin (**1**) in DMSO-d_6. 18
Table S7. NMR data of emestrin B (**2**) in $CDCl_3$. 19
Table S8. NMR data of emestrin F (**3**) in $CDCl_3$. 20
Table S9. NMR data of emestrin M (**4**) in DMSO-d_6. 21
Table S10. NMR data of emestrin J (**5**) in $CDCl_3$. 22
Table S11. NMR data of emestrin L (**6**) in DMSO-d_6. 23
Supplementary Figures........ 24
Figure S1. Aryl-aryl ether formation in the biosynthesis of natural sources. 24
Figure S2. LC-MS profiles of emestrin (**1**) conversion to emestrin B (**2**) in the presence of NADPH. 25
Figure S3. Target gene deletion and PCR verification for *E. quadrilineata* strains. 26
Figure S4. Phylogenetic tree of 44 cytochrome P450 proteins. 27
Figure S5. Heterologous expression and PCR verification for *A. nidulans* strains. 28
Figure S6. LC-MS analysis of **5a** metabolism by EmeO 29
Figure S7. The ^{1}H NMR spectrum of emestrin (**1**) in DMSO-d_6 (500 MHz). 30
Figure S8. The ^{1}H NMR spectrum of emestrin B (**2**) in $CDCl_3$ (600 MHz). 31
Figure S9. The ^{13}C {^{1}H} NMR spectrum of emestrin B (**2**) in in $CDCl_3$ (150 MHz). 32
Figure S10. The DEPT spectrum of emestrin B (**2**) in $CDCl_3$ (150 MHz). 33
Figure S11. The ^{1}H-^{1}H COSY spectrum of emestrin B (**2**) in $CDCl_3$. 34
Figure S12. The HSQC spectrum of emestrin B (**2**) in $CDCl_3$. 35
Figure S13. The HMBC spectrum of emestrin B (**2**) in $CDCl_3$. 36
Figure S14. The ^{1}H NMR spectrum of emestrin F (**3**) in $CDCl_3$ (500 MHz). 37
Figure S15. The ^{13}C {^{1}H} NMR spectrum of emestrin F (**3**) in in $CDCl_3$ (125 MHz). 38
Figure S16. The HMBC spectrum of emestrin F (**3**) in $CDCl_3$. 39
Figure S17. The ^{1}H NMR spectrum of emestrin M (**4**) in DMSO-d_6 (500 MHz). 40
Figure S18. The ^{13}C {^{1}H} NMR spectrum of emestrin M (**4**) in DMSO-d_6 (125 MHz). 41
Figure S19. The DEPT spectrum of emestrin M (**4**) in DMSO-d_6 (125 MHz). 42
Figure S20. The ^{1}H-^{1}H COSY spectrum of emestrin M (**4**) in DMSO-d_6. 43

Figure S21. The HSQC spectrum of emestrin M (**4**) in DMSO-d_6. 44
Figure S22. The HMBC spectrum of emestrin M (**4**) in DMSO-d_6. 45
Figure S23. The ^{1}H NMR spectrum of emestrin J (**5**) in $CDCl_3$ (500 MHz). 46
Figure S24. The ^{1}H NMR spectrum of emestrin L (**6**) in DMSO-d_6 (500 MHz). 47
Figure S25. The ^{13}C {^{1}H} NMR spectrum of emestrin L (**6**) in DMSO-d_6 (125 MHz). 48
Figure S26. The DEPT spectrum of emestrin L (**6**) in DMSO-d_6 (125 MHz). 49
Figure S27. The ^{1}H-^{1}H COSY spectrum of emestrin L (**6**) in DMSO-d_6. 50
Figure S28. The HSQC spectrum of emestrin L (**6**) in DMSO-d_6. 51
Figure S29. The HMBC spectrum of emestrin L (**6**) in DMSO-d_6. 52
Figure S30. The UV spectra of compounds **1−6** in ACN. 53
Figure S31. Isotopic pattern of $[M+H]^+$ or $[M+Na]^+$ ions of compounds **1−6**. 54
Figure S32. The experimental ECD spectra of compounds **1−6** in ACN. 55
Reference 56

Experimental Procedures

General experimental procedures

The experimental Electronic Circular Dichroism (ECD) and UV spectra were measured on a J-1500 CD spectrometer (Jasco Deutschland GmbH, Pfungstadt, Germany). The samples were dissolved in acetonitrile (ACN) and measured in the range of 190–400 nm by using a 1 mm path length quartz cuvette (Hellma Analytics, Müllheim, Germany). The polarimeter Jasco DIP-370 was employed to ascertain the optical rotation at 20°C using the D-line of the sodium lamp (λ=589.3 nm). Prior to the measurement, the polarimeter was calibrated with ACN as solvent. A XtaLAB Synergy R, HyPix diffractometer was used for crystal X-ray diffraction data measurement (Rigaku, Takatsuki, Japan). ^{1}H, ^{13}C and 2D NMR spectra were obtained from an ECZ-400S and an ECA-500 MHz (JEOL, Akishima, Tokyo, Japan) and a Bruker AV-600 spectrometer (Bruker instrument, Inc, Zurich, Switzerland) in DMSO-d_6 or $CDCl_3$. LC-MS data were recorded on an Agilent HPLC series 1260 (Agilent Technologies) system equipped with a Bruker microTOF QIII mass spectrometer (Bruker Daltonik, Germany) by using a VDSpher PUR 100 C18-M-SE column (150 × 2 mm, 3 μm) (VDS optilab Chromatographie Technik, Montabaur, Germany) with a gradient of 5–100% ACN/H_2O in 30 min and 100% ACN/H_2O for additional 5 min, if necessary, and a flow rate at 0.3 mL/min. Exact mass in the range of m/z 100–1500 was measured in positive ESI mode with a capillary voltage of 4.5 kV and collision energy of 8.0 eV and calibrated with sodium formate. The software Compass DataAnalysis 4.2 was utilized for data processing. An Agilent HPLC series 1200 equipped with a VDSpher PUR 100 C18-M-SE column (250 × 10 mm, 5 μm) and gradients of ACN in H_2O at a flow rate of 2.0 mL/min was used for HPLC purification of the described compounds. Silica gel 60 (400–230 mesh, Carl Roth GmbH + Co., Karlsruhe, Germany) was used for column chromatography.

Fungal strain and genome sequencing

Emericella quadrilineata ACCC 31557 was purchased from the Agricultural Culture Collection of China (ACCC). Genomic DNA for sequencing was extracted according to a published protocol.[1] Sequencing was performed on an Illumina NovaSeq PE150 platform by Beijing Novogene Bioinformatics Technology Co., Ltd. The raw data were assembled with SOAP denovo software version 2.04.

Extraction of genomic DNA for genetic manipulation

E. quadrilineata ACCC 31557 and *Aspergillus nidulans* strains (Table S3) were cultivated in 3 mL PDB medium (Carl Roth GmbH + Co., Karlsruhe, Germany) and LMM medium (10 g/L glucose, 50 mL/L nitrate salt solution, and 1 mL/L trace element solution) with necessary nutritional supplements at 37°C for 3 days, respectively. Fresh mycelium was dried on filter paper and crushed with the help of liquid nitrogen. The powder was transferred to 200 µL LETS buffer (20 mM EDTA, 10 mM Tris-HCl, 0.5% SDS, 0.1 M LiCl-H_2O, pH 8.0) and crushed with 5 glass beads in a MiniLys homogeniser (Bertin Technologies, Montigny-le-Bretonneux, France) for 240 seconds at full speed. The cell lysate was extracted with 200 µL phenol/chloroform/isoamyl alcohol (25:24:1) for twice. The aqueous phase was then transferred into 300 µL cold absolute ethanol and centrifuged at 13000 rpm and 4°C for 20 min. The pellet was washed with 500 µL 70% ethanol, dried at 55°C for 15 min, and dissolved in distillated H_2O.

Polymerase chain reaction (PCR)

A T100 Thermal Cycler (BioRad, Hercules, America) was used for polymerase chain reaction. Components and protocols of Q5 Hot Start High-Fidelity DNA polymerase from New England Biolabs (NEB, Ipswich, America) and Phusion polymerase were used for PCR amplification. The primers were synthesized by Seqlab GmbH (Göttingen, Germany) and are listed in Table S4.

Construction of plasmids

Gene deletion and heterologous expression plasmids were constructed *via* homologous recombination in *E. coli* DH5α (Table S5), which was cultivated in liquid lysogeny broth (LB, 10 g/L tryptone, 5 g/L yeast extract, 10g/L NaCl) or on LB plates (with 15 g/L agar) at 37°C with ampicillin (100 µg/mL) for selection.[2]

For gene deletion experiments, the split marker strategy was employed. The upstream and downstream regions of approximately 1.5 kbp of the target genes *emeP, emeO*, *emeE,* and *emeR* were amplified from *E. quadrilineata* ACCC 31557 genomic DNA and ligated into the NotI/BglII-linearized p5HY[3] vector and SalI/HindIII-linearized p3YG[3] vector by using homologous recombination in *E. coli*.[2]

Heterologous expression plasmids for *A. nidulans* LO8030 were constructed by PCR amplification of the target sequence from *E. quadrilineata* ACCC 31557 genomic DNA and

cloned into the SfoI-linearized vector pJN017,[4] which contains the flanking region of the *wA* gene, the *gpdA* promoter, and the *afribo* selection marker. The amplified sequences were inserted between the *gpdA* promoter and *afribo* marker using 20–22 bp overlaps compatible to the SfoI restriction site.

Fungal transformation

Creation of deletion strains in *E. quadrilineata* and heterologous expression in the recipient host *A. nidulans* were performed with polyethylene glycol (PEG)-mediated protoplast transformation. The spores of *E. quadrilineata* were collected from rice medium (5g rice and 5 mL H_2O) after cultivation at 25°C for 20 days followed by storage at 4°C for 2 days. The spores of *A. nidulans* LO8030 were collected from GMM medium (LMM with 1.6% agar) after cultivation at 37°C for 5 days. The spores were inoculated in a 250 mL flask containing 30 mL LMM medium supplied with UURP (1.2 g/L uridine (U), 1.0 g/L uracil (U), 1 mg/L pyridoxine (P), and 1 mg/L riboflavin (R)). *E. quadrilineata* ACCC 31557 and *A. nidulans* LO8030 were cultivated at 180 rpm, at 37 and 33°C, respectively.

For protoplastation, the germlings were harvested by centrifugation in 15 mL falcons at 5000 rpm for 10 min, washed with 3 mL osmotic solution (1.2 M $MgSO_4$, 10 mM sodium phosphate buffer). The harvested mycelia were transferred to a sterile 50 mL flask containing 10 mL lysing solution [200 µL of cellulase TXL, 40 µL chitinase, 8 µL protease (all from ASA Spezial Enzyme GmbH, Wolfenbüttel, Germany) and 20 mg yatalase from *Corynebacterium* sp. OZ-21 (OZEKI Co., Ltd.) dissolved in 10 mL of osmotic solution]. The mixture was kept at 37°C, 100 rpm for 2.5 hours. The obtained protoplasts were then collected using 10 mL trapping buffer (0.6 M sorbitol, 0.1 M Tris-HCl, pH 7.0) and centrifuged at 5000 rpm at 4°C for 8 min. The protoplast layer was separated by carefully transferring into a 15 mL falcon tube and then washing twice with cold STC buffer (1.2 M sorbitol, 10 mM $CaCl_2$, and 10 mM Tris-HCl, pH 7.5). The final protoplasts were resuspended in 300 µL cold STC buffer.

For transformation, 100 µL of the protoplast suspension (approximately 5.0×10^7/mL) were incubated with 5–10 µg of purified DNA fragments on ice for 50 min. 1.25 mL of PEG solution (60% PEG 4000, 50 mM $CaCl_2$, 50 mM Tris-HCl, pH 7.5) were added, gently swirled, and then incubated at room temperature for 25 min. 4.75 mL of STC buffer were then added to the mixture, followed by gentle mixing and spreading onto 6 plates with SMM bottom medium (1.0% glucose, 50 mL/L 20x nitrate salt solution, 1 mL/L trace element solution, 1.2 M sorbitol, and 1.6% agar), kept at room temperature for 3 h. The plates were overlaid with

SMM top medium (SMM bottom medium with 0.8% agar) and incubated at 37°C for 3–5 days. For gene deletion in *E. quadrilineata* ACCC 31557, both bottom and top agar media were supplemented with 200 µg/mL hygromycin B for selection. Hygromycin B-resistant fungal colonies were transferred onto fresh PDA plates containing 200 µg/mL hygromycin B for further selection. For heterologous expression in *A. nidulans* LO8030, media were supplemented with UUP (without R) for selection. *A. nidulans* colonies were transferred onto fresh GMM supplemented with UUP for further selection. Diagnostic PCR amplification was used to verify the resulted deletion mutants *E. quadrilineata* YC1 (Δ*emeP*), YC3 (Δ*emeO*), YC4 (Δ*emeE*), and YC5 (Δ*emeR*). The expression plasmids pYC17 (*emeO*), pYC16 (*emeE*), and pYC15 (*emeR*) were introduced into *A. nidulans* LO8030, resulting in the *A. nidulans* strains YC101, YC102, and YC103, respectively.

Fermentation, extraction and isolation.

Emestrin (**1**) and emestrin B (**2**) were isolated from the fermentation extracts of *E. quadrilineata* ACCC 31557, while emestrin F (**3**), emestrin M (**4**), and emestrin J (**5**) were obtained from the fermentation extracts of *E. quadrilineata* YC4, YC5, and YC3, respectively. The strains were cultivated on PDA medium plate at 25°C for 7 days. The mycelia were inoculated then into 500 mL Erlenmeyer flask containing 100 mL PDB medium for seed culture at 25°C for 3 days on a rotary shaker set at 180 rpm. For metabolite isolation, the fungal strains were cultivated in rice medium at 25°C for 14 days. In order to increase the product yield of emestrin J (**5**), 4% GSH (vitalundfitmit100 GmbH, Jüchen, Germany) was added to the rice medium.

The cultures underwent a twice extraction with EtOAc. The combined extracts were subjected to evaporation to remove all the solvent. 20 mL of petroleum ether (60–80°C) were then added, gently swirled. The supernatant was discarded. The residue was extracted three times with 100 mL CH_2Cl_2 and mixed with silica gel for further separation on a silica gel column. Fractionation was achieved by step gradients with petroleum ether-acetone from 10:1, 5:1, and 2:1. All the compounds of interest, *i.e.* emestrin (**1**), emestrin B (**2**), emestrin F (**3**), emestrin M (**4**), and emestrin J (**5**) were found in the fractions with 2:1 petroleum ether-acetone, which were further purified on a semi-preparative HPLC using 55, 55, 60, 65, and 65% ACN-H_2O as elution solvents, affording **1** (3.3 mg), **2** (2.7 mg), **3** (33.9 mg), **4** (82.5 mg), and **5** (281.3 mg), respectively.

Emestrin L (**6**) was isolated from the *A. nidulans* YC101 culture after feeding with emestrin J (**5**). *A. nidulans* YC101 was cultivated in a 500 mL Erlenmeyer flask containing 100 mL

LMM medium supplemented with UUPR for seed culture at 25°C for 3 days on a rotary shaker set at 180 rpm. The strain was then inoculated into 2 L LMM medium with UUPR at 25°C for 2 days on a rotary shaker set at 200 rpm. Afterward, **5** was added into the culture for another 2 days. The fungal cultures were extracted twice with EtOAc, separated through a silica gel column as mentioned above. Further purification on the semi-preparative HPLC using 65% ACN-H_2O afforded **6** (2.7 mg).

Heterologous expression and feeding experiments

To prove the gene function by heterologous expression and feeding experiments, the fungal strains were first cultivated in 30 mL LMM medium with UUP at 25°C for 2 days on a rotary shaker set at 200 rpm. The medium was replaced by 5 mL fresh LMM media with UUP, followed by addition of 0.2 mg of the respective precursor in 2 µL DMSO. After further cultivation for 2 days at 25°C and 200 rpm, 1 mL culture containing both mycelia and medium was first separated by centrifugation and extracted with EtOAc parallelly. The extracts were combined, evaporated, and dissolved in MeOH for LC-MS analysis, and dissolved in methanol for LC-MS analysis. *A. nidulans* BK06[5] was used as the control strain for feeding experiments.

Microsome preparation and *in vitro* assays of 3–5 for EmeE, EmeR, and EmeO

The *A. nidulans* transformants containing *emeE*, *emeR*, and *emeO* were cultivated in 50 mL LMM liquid medium with UUP at 25°C for 2 days. The fresh mycelia were grounded in liquid nitrogen, suspended in 4 mL buffer containing 50 mM Tris-HCl, 50 mM NaCl, and 5% glycerol (pH 7.5), and centrifuged at 4°C, 4000 rpm for 20 min to remove cellular debris. The supernatant was taken for further centrifugation at 4°C, 13,300 rpm for 1 h. Afterward, the pellets were resuspended in 100 µL of the same buffer to afford the microsomal fractions. Compounds **3**, **4**, and **5** (0.1 mM) were incubated with 100 µL microsomal fraction of EmeE, EmeR, and EmeO, in the presence of NADPH (10 mM) at 25°C for 2 h, respectively. The reaction mixtures were then extracted with 500 µl EtOAc. The organic phases were evaporated to dryness and dissolved in ACN for LC-MS analysis. Microsomes from *A. nidulans* BK06[5] were used as a negative control.

Conversion of 1 to 2 in the presence of NADPH

A 28 mM stock solution of compound **1** was prepared in DMSO and diluted with distilled water to 28 µM. After addition of solid NADPH to a final concentration of 10 mM, 1 mL

mixtures were incubated at 25°C and 100 rpm for 1, 2, and 4 h. After incubation, the reaction mixtures were extracted with 1 mL EtOAc, concentrated, and dissolved in 200 µL methanol for LC-MS analysis. Incubations without NADPH served as controls.

X-ray diffraction analysis

Single crystals of emestrin B (**2**) were obtained using the slow evaporation from CH_2Cl_2-EtOH-H_2O (100:95:5) at room temperature. A suitable crystal was selected and analysis on a ROD, Synergy Custom DW system, HyPix diffractometer. The crystal was kept at 100 Kelvin during data collection. Using Olex2,[6] the structure was solved with the SHELXS[7] structure solution program using direct methods and refined with the SHELXL[8] refinement package using Least Squares minimisation. Crystal data see Table S1.

Structural elucidation

The structures of the isolated compounds were elucidated by analysis of their spectroscopic data (Tables S6–S11, Figures S7–S32). The known compounds **1**–**3** and **5** were identified by comparison of their MS and ^{1}H-NMR data with those of emestrin, emestrin J, emestrin F and emestrin B reported in the literature.[9–12] The new compounds **4** and **6** were determined by extensive analysis of spectroscopic data, including those of ECD, ^{13}C NMR, ^{1}H-^{1}H COSY, HSQC, and HMBC.

The ^{1}H NMR spectra of all the compounds (**1**–**6**) showed signals of two methyl groups attached to hetero atoms, at δ_H 3.93–4.07 (OCH$_3$-4′, s) and δ_H 2.99–3.54 ppm (NCH$_3$-2, s), a disubstituted double bond with resonances at δ_H 4.68–5.01 (H-8) and 6.32–6.42 (H-9), a trisubstituted double bond at δ_H 6.67–7.05 (H-11), as well as four sp^3-hybridized methines or methylenes at δ_H 3.14–5.84 (H-6, H-7, H13, and H-7″). Further analysis revealed the presence of signals for two 1,3,4-trisubstituted benzene rings at δ_H 6.81–8.72 in those of **1**–**4** and **6**, whereas emestrin J (**5**) displayed only one 1,3,4-trisubstituted benzene ring with signals at δ_H 7.67 (1H, d), 7.72 (1H, dd), and 6.89 ppm (1H, d) along with a monosubstituted benzene ring at δ_H 7.22 (2H, br d), 7.09 (2H, br t), and 7.17 (1H, br t). In ECD spectra, **1**, **2**, **5**, and **6** showed similar cotton effects, while **3** and **4** slightly different from other compounds (Figure S32). Together with the typical UV spectra (Figure S30), these compounds were identified to be emestrin (**1**)[9] and congeners (**2**–**6**).

The molecular formula of **2** was deduced to be $C_{27}H_{22}N_2O_{10}S_3$ based on its [M+Na]$^+$ at *m/z* 653.0307, one sulfur atom more than that of **1**. The ^{1}H and ^{13}C NMR data (Figures S8 and S9) of **2** are very similar to those of **1**, indicating the presence of a trisulfide bridge in **2**

instead of a disulfide sulfur bridge in **1**. These data are consistent with those of emestrin B reported in the literature.[11] After confirmation of the structure by X-ray (Table S1, accession number CCDC 2488010), we reassigned the chemical shifts in ^{13}C NMR spectrum based on 2D NMR spectra (Table S7).

Emestrin F (**3**) with a [M+Na]$^+$ ion at *m/z* 605.0667 and a molecular formula of $C_{27}H_{22}N_2O_9S_2$, has one oxygen atom less than that of **1**. ^{1}H NMR spectrum (Table S8, Figure S14) showed that the signals of oxymethine (H-7″ and OH-7″) in **1** were replaced by two doubles at δ_H 3.47 and 3.96 ppm with a coupling constant of 13.4 Hz. HMBC correlations (Figure S16) from H-7″ to C-3, C-4, C-2″, and C-6″ proved a methylene group at C-7″. Therefore, **3** was determined to be emestrin F,[12] a 7″-deoxy analogue of emestrin (**1**).

Compound **4** has the same molecular formula as **3** based on its [M+H]$^+$ ion at *m/z* 583.0846 (calcd for $C_{27}H_{23}N_2O_9S_2^+$, 583.0839). In the ^{1}H NMR spectrum (Table S9, Figure S17) of **4**, the signals of the oxymethine at C-13 (H-13 and OH-13) of **1** were replaced by two doubles at δ_H 3.19 and 3.88 ppm with a coupling constant of 18.0 Hz. In addition, the chemical shift of C-13 in the ^{13}C NMR spectrum (Figure S18) was upfield shifted by $\Delta\delta_C$ −40.3 and −45.3 ppm, in comparison with those of **1**[9] and **2,** respectively. DEPT-135° spectrum (Figure S19) also confirmed the methylene character of C-13. The HMBC correlations (Figure S22) from H_2-13 to C-11, C-12, and C-14 further supported **4** as a 13-deoxy congener of emestrin (**1**), termed emestrin M in this study.

Emestrin J (**5**) has a molecular formula of $C_{27}H_{24}N_2O_7S_2$, which is deduced from its [M+Na]$^+$ ion at *m/z* 575.0928 (calcd for $C_{27}H_{24}N_2O_7S_2Na^+$, 575.0917), *i.e*. three oxygen atoms and one degree of unsaturation less than that of **1**. As mentioned above, **5** was equipped with one monosubstituted benzene ring instead of a 1,3,4-trisubstituted benzene ring, indicating the absence of the aryl-aryl ether bond between C-3′ and C-3″ in **1**. In addition, signals of the two sp^3-hybridized methines at C-13 and C-7″ of **1** were replaced by those of two methylene groups CH_2-13 [δ_H 3.14 (H-13α, ddd, *J*=18.4 Hz, *J*=2.4Hz, *J*=1.3Hz) and δ_H 4.12 (H-13β, dt, *J*=17.8 Hz, *J*=1.7Hz)] and CH_2-7″ (δ_H 3.67, s) in the spectrum of **5** (Tables S6 and S10, Figure S23). These data correspond very well to those of emestrin J.[10]

Compound **6** with a molecular formula of $C_{27}H_{22}N_2O_8S_2$ has two oxygen atoms less than **1**. Similar to that **5**, ^{1}H NMR data of **6** (Table S11, Figure S24) also suggested absence of the two oxymethine signals of **1** at C-13 and C-7″. Instead, resonances of two coupling methylene groups with large coupling constants appeared at 3.22 and 3.92 (*J*=17.8 Hz) as well as 3.58 and 3.85 ppm (*J*=13.6 Hz). This conclusion was supported by the ^{13}C NMR and DEPT-135° spectra (Figures S25 and S26). In comparison to those of **1**[9] the chemical shifts

of C-13 and C-7″ in **6** are upfield shifted by Δδ_C −34.3 and −37.6, respectively. The HMBC correlations (Figure S29) from H_2-13 to C-12 and C-14 as well as H_2-7″ to C-3, C-4, C-2″, and C-6″ further confirmed the presence of two CH_2 groups at C-13 and C-7″ in **6**. This compound has not been described prior to this work and is named emestrin L.

Physiochemical properties

Emestrin (1). White amorphous powder; $[\alpha]^{20}_D$ +182.4 (*c* 0.04, ACN). $C_{27}H_{22}N_2O_{10}S_2$, ESI-MS *m/z* 599.0790 $[M+H]^+$ (calcd for $C_{27}H_{23}N_2O_{10}S_2^+$: 599.0789, Figure S31); ^1H NMR (500 MHz, DMSO-d_6) and ^{13}C NMR (125 MHz, DMSO-d_6) spectral data are listed in Table S6 and NMR spectra in Figure S7, respectively. UV and ECD spectra are given in Figures S30 and S32, respectively.

Emestrin B (2). White amorphous powder; $[\alpha]^{20}_D$ +180.0 (*c* 0.05, ACN). $C_{27}H_{22}N_2O_{10}S_3$, ESI-MS *m/z* 653.0308 $[M+Na]^+$ (calcd for $C_{27}H_{22}N_2O_{10}S_3$ Na^+: 653.0329, Figure S31); ^1H NMR (600 MHz, $CDCl_3$) and ^{13}C {^1H} NMR (150 MHz, $CDCl_3$) spectral data are listed in Table S7 and NMR spectra in Figures S8–S13, respectively. UV and ECD spectra are given in Figures S30 and S32, respectively.

Emestrin F (3). White amorphous powder; $[\alpha]^{20}_D$ -220.0 (*c* 0.02, ACN). $C_{27}H_{22}N_2O_9S_2$, ESI-MS *m/z* 605.0661 $[M+Na]^+$ (calcd for $C_{27}H_{22}N_2O_9S_2Na^+$: 605.0661, Figure S31); ^1H NMR (500 MHz, $CDCl_3$) and ^{13}C {^1H} NMR (125 MHz, $CDCl_3$) spectral data are listed in Table S8 and NMR spectra in Figures S14–S16, respectively. UV and ECD spectra are given in Figures S30 and S32, respectively.

Emestrin M (4). White amorphous powder; $[\alpha]^{20}_D$ +300.0 (*c* 0.01, ACN). $C_{27}H_{22}N_2O_9S_2$, ESI-MS *m/z* 583.0848 $[M+H]^+$ (calcd for $C_{27}H_{23}N_2O_9S_2^+$: 583.0839, Figure S31); ^1H NMR (500 MHz, DMSO-d_6) and ^{13}C {^1H} NMR (125 MHz, DMSO-d_6) spectral data are listed in Table S9 and NMR spectra in Figures S17–S22, respectively. UV and ECD spectra are given in Figures S30 and S32, respectively.

Emestrin J (5). White amorphous powder; $[\alpha]^{20}_D$ +0.0 (*c* 0.03, ACN). $C_{27}H_{24}N_2O_7S_2$, ESI-MS *m/z* 575.0929 $[M+Na]^+$ (calcd for $C_{27}H_{24}N_2O_7S_2Na^+$: 575.0917, Figure S31); ^1H NMR (500 MHz, $CDCl_3$) and ^{13}C {^1H} NMR (125 MHz, $CDCl_3$) spectral data are listed in Table S10

and NMR spectra in Figure S23, respectively. UV and ECD spectra are given in Figures S30 and S32, respectively.

Emestrin L (6). White amorphous powder; $[\alpha]^{20}_{D}$ +275.0 (*c* 0.02, ACN). $C_{27}H_{22}N_2O_8S_2$, ESI-MS *m/z* 567.0890 $[M+H]^+$ (calcd for $C_{27}H_{23}N_2O_8S_2^+$: 567.0890, Figure S31); ^1H NMR (500 MHz, DMSO-d_6) and ^{13}C {^1H} NMR (125 MHz, DMSO-d_6) spectral data are listed in Table S11 and NMR spectra in Figures S24–S29, respectively. UV and ECD spectra are given in Figures S30 and S32, respectively.

Supplementary Tables

Table S1. Crystal data and structure of emestrin B (**2**)

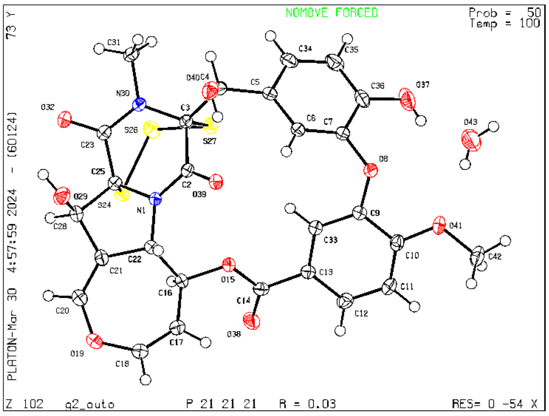

Name	emestrin B	
Identification code	g2_auto	
Empirical formula	$C_{27}H_{23.5}N_2O_{10}S_3$, 0.75($H_2O$)	
Formula weight	644.16	
Temperature	100.00(10) K	
Wavelength	1.54184	
Crystal system	orthorhombic	
Space group	$P2_12_12_1$	
Unit cell dimensions	a = 6.99290(10) Å	α = 90°
	b = 11.26810(10) Å	β = 90°
	c = 34.1807(4) Å	γ = 90°
Volume	2693.33(6) Å^3	
Z	4	
D_{calc}	1.589 g/cm^3	
μ	3.114 mm^{-1}	
F (000)	1334.0	
Crystal size	0.15 × 0.12 × 0.05 mm^3	
Radiation	Cu Kα (λ=1.54184)	
2θ range for data collection	5.17 to 154.886°	
Index ranges	$-6 \le h \le 8$, $-13 \le k \le 13$, $-43 \le l \le 41$	
Reflections collected	24647	
Independent reflections	5397 [R_{int} = 0.0376, R_{sigma} = 0.0274]	
Data/restraints/parameters	5397/0/396	
Goodness-of-fit on F^2	1.055	
Final R indexes [I>=2σ (I)]	R_1 = 0.0300, wR_2 = 0.0791	
Final R indexes [all data]	R_1 = 0.0324, wR_2 = 0.0803	
Largest diff. peak/hole	0.42/-0.31 e Å^{-3}	
Flack parameter	0.007(7)	

Table S2. Similarities and putative functions of proteins encoded by the *eme* cluster in *A. quadrilineata* ACCC 31557 and the *erc* cluster in *Emericella. nidulans* 1454.

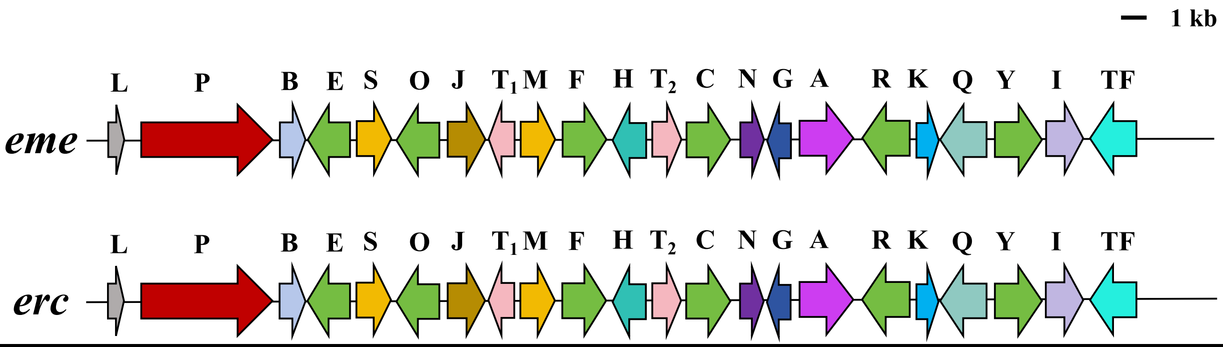

***E. quadrilineata* ACCC 31557**	***E. nidulans* 1454**		**Other strians**		**Putative function**
**Protein (aa)**	**Protein (aa)**	**Identity/% (cover/%)**	**Proteins**	**Identity/% (cover/%)**	
EmeL (189)	ErcL (189)	100.0 (100)	AtaL (155 aa, XP_001212647.1, *A. terreus* NIH2624)	31.6 (96)	hypothetical protein
EmeP (1693)	ErcP (1693)	99.7 (100)	AclP (1625 aa, XP_023089485.1, *A. oryzae* RIB40)	39.8 (93)	NRPS
EmeB (337)	ErcB (337)	100.0 (100)	No known homologous protein	/	putative 3-dehydroshikimate dehydratase
EmeE (547)	ErcE (547)	99.8 (100)	No known homologous protein	/	putative cytochrome P450
EmeS (427)	ErcS (427)	99.5 (100)	GsfB (419 aa, D7PI16.1, *Penicillium aethiopicum*)	42.1 (98)	*O*-methyltransferase
EmeO (516)	ErcO (516)	99.8 (100)	Tri4 (517 aa, G0KYB2.1, *Trichoderma arundinaceum*)	33.4 (97)	cytochrome P450
EmeJ (385)	ErcJ (385)	100.0 (100)	GliJ (388 aa, Q4WMJ8.2, *A. fumigatus* Af293)	55.6 (97)	dipeptidase
EmeT$_1$ (316)	ErcT$_1$ (316)	100.0 (100)	GliT (335 aa, XP_750863.1, *A. fumigatus* Af293)	50.6 (93)	thioredoxin reductase
EmeM (431)	ErcM (431)	100.0 (100)	AclM (433 aa, Q2UPB3.1, *A. oryzae* RIB40)	56.3 (97)	*O*-methyltransferase
EmeF (501)	ErcF (501)	100.0 (100)	GliF (504 aa, Q4WMJ0.1, *A. fumigatus* Af293)	54.5 (98)	cytochrome P450
EmeH (405)	ErcH (405)	99.3 (100)	AtaH (303aa, Q0CS68.1, *A. terreus* NIH2624)	37.2 (93)	*O*-acyl transferase
EmeT$_2$ (306)	ErcT$_2$ (307)	99.7 (100)	GliT (335 aa, XP_750863.1, *A. fumigatus* Af293)	38.8 (93)	thioredoxin reductase
EmeC (513)	ErcC (513)	100.0 (100)	AclC (486 aa, Q2UPB1.1, *A. oryzae* RIB40)	53.9 (95)	cytochrome P450
EmeN (278)	ErcN (278)	100.0 (100)	GliN (282 aa, XP_750861.1, *A. fumigatus* Af293)	35.2 (93)	*N*-methyltransferase
EmeG (252)	ErcG (252)	99.2 (100)	AclG (221 aa, Q2UPB2.1, *A. oryzae* RIB40)	71.4 (100)	glutathione S-transferase
EmeA (583)	ErcA (583)	100.0 (100)	AtaA (449 aa, XP_001212653.1, *A. terreus* NIH 2624)	40.3 (94)	MFS
EmeR (523)	ErcR (523)	100.0 (100)	FtmP450-3 (490 aa, Q4WAX0.1, *A. fumigatus* Af293)	34.1 (89)	cytochrome P450
EmeK (269)	ErcK (269)	100.0 (100)	GliK (273 aa, E9R9Y3.1, *A. fumigatus* Af293)	40.2 (91)	gamma-glutamyl cyclotransferase
EmeQ (563)	ErcQ (563)	99.6 (100)	M9 (541 aa, A0A3G1DJF8.1, *Phoma* sp. MF5453)	48.6 (96)	acyl-CoA ligase
EmeY (509)	ErcY (509)	100.0 (100)	AtaY (228 aa, Q0CS66.1, *A. terreus* NIH2624)	61.1 (45)	cytochrome P450
EmeI (442)	ErcI (442)	100.0 (100)	AclI (420 aa, Q2UPB9.1, *A. oryzae* RIB40)	37.7 (99)	C-S lyase
EmeTF (418)	ErcTF (428)	99.8 (100)	No known homologous protein	23.7 (97)	putative zinc finger transcription factor

A.: *Aspergillus*

The reported sequences are deposited in GenBank under the accession numbers PX410941 (*eme* cluster) and PX417294 (*erc* cluster).

Table S3. Fungal strains created and used in this study.

**Strain name**	**Description**	**Source/Reference**
E. quadrilineata ACCC 31557	wildtype	ACCC
E. quadrilineata YC1	Δ*emeP* in ACCC 31557	this study
E. quadrilineata YC3	Δ*emeO* in ACCC 31557	this study
E. quadrilineata YC4	Δ*emeE* in ACCC 31557	this study
E. quadrilineata YC5	Δ*emeR* in ACCC 31557	this study
A. nidulans LO8030	heterologous expression host	[13]
A. nidulans BK06	Δ*wA*::*gpdA*(p)-*Afribo* in LO8030	[5]
A. nidulans YC101	Δ*wA*::*gpdA*(p): *emeO* -*Afribo* in LO8030	this study
A. nidulans YC102	Δ*wA*::*gpdA*(p): *emeE* -*Afribo* in LO8030	this study
A. nidulans YC103	Δ*wA*::*gpdA*(p): *emeR* -*Afribo* in LO8030	this study

Table S4. Primers used in this study.

Primer name	Primer sequence 5'-3'	Used for
prYC1	AAGAATTGTTAATTAAGAGCTCAGATCTGTGATAGTTGCGAAGAGTA	*emeP* deletion
prYC2	CCTCACTAAAGGGCGGCCGCACTAGGAGGGCGGTTTCAAGAAAAG	
prYC3	ACTCACTATAGGGCCCGGGCGTCGATGATCTCGAGGATCTGGAAG	
prYC4	CTTAGCTAGCCGCGGTACCAAGCTTCACAGCGTAGGATAACGAGT	
prYC5	AAGAATTGTTAATTAAGAGCTCAGATCGCTATTCTACGTGCCTTGG	*emeE* deletion
prYC6	CCTCACTAAAGGGCGGCCGCACTAGCCAGGTCATGCAACTCAAGG	
prYC7	ACTCACTATAGGGCCCGGGCGTCGACAATGGTTGCAATTGCCAGG	
prYC8	CTTAGCTAGCCGCGGTACCAAGCTTTCGACAGGTCCATAAGCCTG	
prYC9	AAGAATTGTTAATTAAGAGCTCAGATCACTTGCAAGCACTACCTTGC	*emeO* deletion
prYC10	CCTCACTAAAGGGCGGCCGCACTAGAGACAGCAGCTATATGGACG	
prYC11	ACTCACTATAGGGCCCGGGCGTCGAGACGACACAATGTCTGGGAA	
PrYC12	CTTAGCTAGCCGCGGTACCAAGCTTCTCTAACATCCCGTCAAAGT	
prYC13	AAGAATTGTTAATTAAGAGCTCAGATCTGGTCTCCTTACCTTTATGC	*emeR* deletion
prYC14	CCTCACTAAAGGGCGGCCGCACTAGGTTACCTGAAAGGGCCTGAG	
prYC15	ACTCACTATAGGGCCCGGGCGTCGAAAGTCGGTGGGCTTGTGTTG	
prYC16	CTTAGCTAGCCGCGGTACCAAGCTAGGCATGTGCCTTGTCAATG	
prYC17	TTGGGGAATTCAGCGAGAGC	*emeP*, *emeO*, *emeE*, and *emeR* deletion
prYC18	TCGAAGTAGCGCGTCTGC	
prYC19	CCGCTTGAGCAGACATCACCACATCTTTCCTTGACATGATGG	heterologous expression and verification of *emeR*
prYC20	CACCATATTTTAATCCCATGTGGATCAGGTGTTCTATGCTGC	
prYC21	CCGCTTGAGCAGACATCACCATGCTAGTGCCGTTATTCTTGGCCAT TCCACTTGCATGC	heterologous expression and verification of *emeE*
prYC22	CACCATATTTTAATCCCATGTGCTATGCGGATCCTGAAGCTG	
prYC23	CCGCTTGAGCAGACATCACCATGCTGAGTATTACAGCCCT	heterologous expression and verification of *emeO*
prYC24	CACCATATTTTAATCCCATGTGCAGGCTTATGGACCTGTCAA	
prYC25	CCAGGAGATGTTGCTGAAGTCG	verification of Δ*emeP*, Δ*emeO*, Δ*emeE*, and Δ*emeR* mutants
prYC26	CGCATTGGTCTTGACCAACTC	
prYC27	CTCTTGGTAGAGCGGACCAG	verification of Δ*emeP* mutant
prYC28	ATGTCCACCTCCATAGCGTT	
prYC29	GTCAGTGAATGGGTGAAGCA	
prYC30	CTGGAATACGACACGGCTTG	verification of Δ*emeE* mutant
prYC31	CAACCTCAAACCTGCCATCC	
prYC32	ACATCCGACCCTTTGCGATG	
prYC33	CATTCCACTTGCATGCGT	
prYC34	TATCGTGGACCTCATCACAC	verification of Δ*emeO* mutant
prYC7	ACTCACTATAGGGCCCGGGCGTCGACAATGGTTGCAATTGCCAGG	
prYC35	CTTTGATGTGGACCTCGTCC	
prYC36	ATGCTGAGTATTACAGCACTCGCAGCCCTAAGCGCAGCCG	
prYC37	GAAGGCCATAGAGCTCGACC	verification of Δ*emeR* mutant
prYC38	CTCGCCTGCTGAGCTTGAAG	
prYC39	TTCAGGTAACTCAGGCCATG	
prYC40	CGTTGGTACTGCGATCGTAA	

The letters in red indicate overlapping sequences for homologous recombination

Table S5. Plasmids constructed in this study.

Plasmid	Insert	Primer 1	Primer 2	Template	T_A	Vector
pYC1	1428 bp upstream of *emeP*	prYC1	prYC2	genomic DNA	55℃	p5HY[3]
pYC2	1492 bp downstream of *emeP*	prYC3	prYC4	genomic DNA	55℃	p3YG[3]
pYC5	1655 bp downstream of *emeE*	prYC5	prYC6	genomic DNA	55℃	p5HY
pYC6	1463 bp upstream of *emeE*	prYC7	prYC8	genomic DNA	55℃	p3YG
pYC7	1549 bp downstream of *emeO*	prYC9	prYC10	genomic DNA	57℃	p5HY
pYC8	1492 bp upstream of *emeO*	prYC11	prYC12	genomic DNA	55℃	p3YG
pYC11	1521 bp downstream of *emeR*	prYC13	prYC14	genomic DNA	55℃	p5HY
pYC12	1488 bp upstream of *emeR*	prYC15	prYC16	genomic DNA	55℃	p3YG
pYC15	*emeR*	prYC19	prYC20	genomic DNA	55℃	pJN017[4]
pYC16	*emeE*	prYC21	prYC22	genomic DNA	55℃	pJN017
pYC17	*emeO*	prYC23	prYC24	genomic DNA	58℃	pJN017

Primers used for PCR amplification are listed in Table S3.

Table S6. NMR data of emestrin (**1**) in DMSO-d_6.

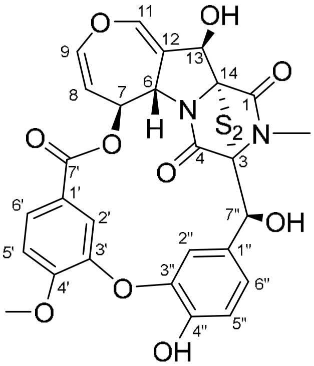

Position	δ_H mult. (*J* in Hz)	Position	δ_H mult. (*J* in Hz)
NCH_3-2	3.24, s	OCH_3-4′	3.93, s
6	5.66 dd (7.4, 2.6)	5′	7.20, d (8.6)
7	4.67, dt (7.4, 2.4)	6′	7.56, dd (8.6, 2.0)
8	4.91, dd (8.4, 2.0)	2″	7.76, d (2.1)
9	6.41, dd (8.4, 2.7)	OH-4″	9.73, s
11	7.05, d (2.6)	5″	6.87, d (8.2)
13	4.96, d (4.2)	6″	7.14, dd (8.2, 2.1)
OH-13	5.95, d (4.2)	7″	5.45, d (7.1)
2′	7.37, d (2.0)	OH-7″	6.22, d (7.1)

The ^{1}H NMR data of **1** correspond well to those of emestrin.[9]

Table S7. NMR data of emestrin B (**2**) in $CDCl_3$.

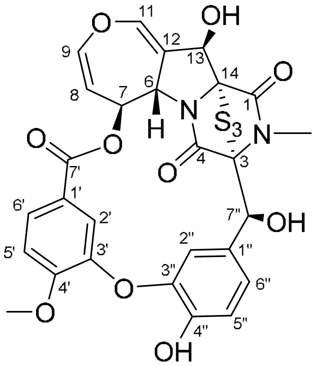

Position	δ_H mult. (J in Hz)	δ_C, type	Key HMBC correlations
1		168.2, C	
NCH_3-2	3.54, s	28.8, CH_3	C-1, C-3
3		78.0, C	
4		165.5, C	
6	5.39, dd (8.5, 2.3)	59.0, CH	C-7, C-8, C-11, C-14
7	5.33, dt (8.5, 2.5)	74.3, CH	C-6
8	5.01, dd (8.5, 2.3)	109.8, CH	C-6, C-7, C-9
9	6.37, dd (8.0, 2.2)	139.0, CH	C-7, C-8, C-11
11	6.83, d (2.4)	143.0, CH	C-6, C-9, C-12, C-13
12		108.3, C	
13	4.81, s	79.8, CH	C-6, C-11, C-12, C-14
14		83.2, C	
1′		122.6, C	
2′	8.36, d (2.0)	123.2, CH	C-1′, C-3′, C-4′, C-6′, C-7′
3′		147.3, C	
4′		154.5, C	
OCH_3-4′	4.07, s	56.5, CH_3	C-4′
5′	7.03, d (8.6)	112.0, CH	C-1′, C-3′, C-4′
6′	7.87, dd (8.6, 2.0)	127.6, CH	C-2′, C-4′, C-7′
7′		165.2, C	
1′′		127.0, C	
2′′	8.72, d (2.2)	126.8, CH	C-3′′, C-4′′, C-6′′, C-7′′
3′′		146.5, C	
OH-4′′	7.01, s		
5′′	6.81, d (8.3)	114.4, CH	C-1′′, C-3′′, C-4′′, C-6′′
6′′	6.93, dd (8.3, 2.2)	130.3, CH	C-2′′, C-4′′, C-5′′, C-7′′
7′′	5.23, br s	76.6, CH	C-3, C-4, C-2′′, C-6′′

The ^1H NMR and ^{13}C NMR data of **2** are consistent with those of emestrin B reported in the literature.[11] We reassigned the ^{13}C NMR chemical shifts using 2D NMR data.

Table S8. NMR data of emestrin F (**3**) in $CDCl_3$.

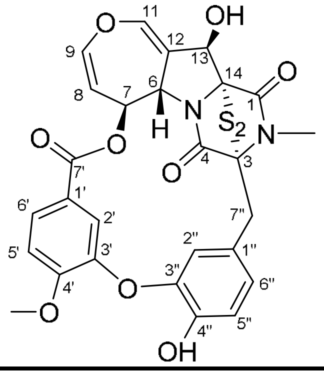

Position	δ_H mult. (*J* in Hz)	δ_C, type	Key HMBC correlations
1		167.1, C	
NCH_3-2	3.22, s	27.8, CH_3	C-1, C-3
3		78.6, C	
4		163.1, C	
6	5.63 ddd (8.0, 2.4, 1.0)	60.7, CH	C-7, C-12, C-14
7	4.92 dt (8.0, 2.3)	75.1, CH	C-6
8	4.92, dd (8.2, 2.5)	107.9, CH	C-6
9	6.33, dd (8.2, 2.4)	138.6, CH	C-7, C-8, C-11
11	6.90, br d (2.4)	142.9, CH	C-13
12		112.9, C	
13	5.01, br s	77.2, CH[a]	C-6, C-11, C-12
OH-13	5.57, d (1,7)		
14		72.9, C	
1′		122.7, C	
2′	7.77, d (2.0)	121.7, CH	C-4′, C-6′
3′		145.5, C	
4′		154.0, C	
OCH_3-4′	4.02, s	56.2, CH_3	C-4′
5′	7.02, d (9.1)	112.0, CH	C-1′, C-3′
6′	7.76, dd (9.1, 2.0)	126.3, CH	C-2′
7′		165.4, C	
1″		123.1, C	
2″	7.90, d (2.1)	125.9, CH	C-4″, C-6″, C-7″
3″		144.5, C	
4″		148.1, C	
OH-4″	6.25, s		C-5″
5″	6.92, d (8.2)	115.3, CH	C-1″, C-3″, C-4″
6″	7.07, dd (8.2, 2.1)	127.8, CH	C-2″, C-4″
7″	3.47, d (13.4)	36.2, CH_2	C-3, C-4, C-2″, C-6″
	3.96, d (13.4)		

[a]Overlapping with the solvent signal, deduced from HMBC.
The ^{1}H NMR data of **3** correspond well to those of emestrin F.[12] We reassigned the ^{13}C {^{1}H} NMR chemical shifts in $CDCl_3$ using 2D NMR data.

Table S9. NMR data of emestrin M (**4**) in DMSO-d_6.

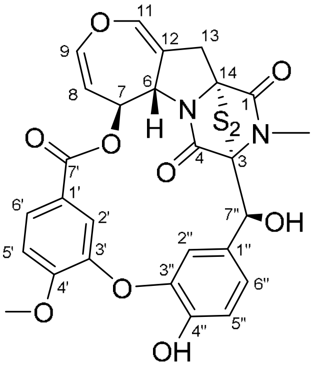

Position	δ_H mult. (*J* in Hz)	δ_C, type	Key HMBC correlations
1		165.8, C	
NCH_3-2	3.30	27.8, CH_3	C-1, C-3
3		79.6, C	
4		160.8, C	
6	5.31, dd (8.4, 2.0)	61.6, CH	C-7, C-12
7	4.83, dt (8.4, 2.0)	73.1, CH	C-8, C-7′
8	4.81, dd (8.4,1.8)	105.4, CH	C-6, C-9
9	6.42, dd (8.4, 2.4)	140.1, CH	C-7, C-8, C-11
11	6.82, br s	138.7, CH	C-6, C-9, C-12
12		113.1, C	
13	3.19, d (18.0)<br>3.88, d (18.0)	34.5, CH_2	C-11, C-12, C-14
14		72.8, C	
1′		122.5, C	
2′	7.47, d (2.0)	119.1, CH	C-3′, C-4′, C-6′, C-7′
3′		145.8, C	
4′		153.5, C	
OCH_3-4′	3.93, s	56.0, CH_3	C-4′
5′	7.18, d (8.4)	112.6, CH	C-1′, C-3′, C-4′
6′	7.51, dd (8.4, 2.0)	124.3, CH	C-2′, C-4′, C-7′
7′		165.3, C	
1″		127.1, C	
2″	8.09, d (2.1)	124.3, CH	C-4″, C-6″, C-7″
3″		143.8, C	
4″		149.5, C	
OH-4″	9.57, s		C-3″, C-4″
5″	6.83, d (8.3)	115.2, CH	C-1″, C-3″, C-4″
6″	7.19, dd (8.3, 2.4)	126.1, CH	C-2″, C-4″
7″	5.48, d (7.8)	73.5, CH	C-3, C-4, C-1″, C-6″
OH-7″	6.21, d (7.8)		C-3, C-7″

Table S10. NMR data of emestrin J (**5**) in $CDCl_3$.

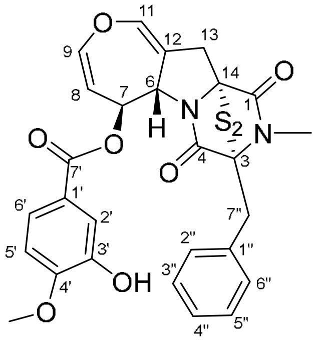

5

Position	δ_H mult. (J in Hz)
NCH_3-2	2.99, s
6	5.34 ddt (8.8, 2.2, 2.0)
7	5.84, dt (8.7, 2.0)
8	4.68, dd (8.2, 1.8)
9	6.32, dd (8.2, 2.1)
11	6.67, td (2.1, 2.0)
13	3.14, dt (18.4, 1.7)
	4.12, ddd (18.4, 2.4, 1.2)
2′	7.67, d (2.1)
OH-3′	5.83, br s
OCH_3-4′	3.96, s
5′	6.89, d (8.5)
6′	7.72, dd (8.5, 2.1)
2″/6″	7.22, br d (8.0)
3″/5″	7.09, br t (7.4)
4″	7.17, br t (7.4)
7″	3.67, s

The ^{1}H NMR data of **5** correspond well to those of emestrin J.[10]

Table S11. NMR data of emestrin L (**6**) in DMSO-d_6.

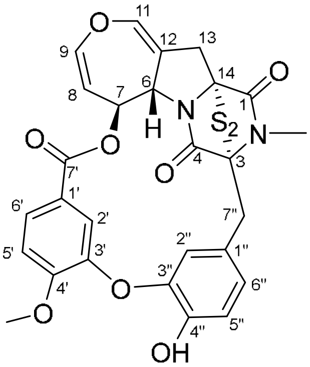

Position	δ_H mult. (J in Hz)	δ_C, type	Key HMBC correlations
1		165.6, C	
NCH_3-2	3.15, s	27.9, CH_3	C-1, C-3
3		78.8, C	
4		162.9, C	
6	5.20, br d (8.7)	61.8, CH	
7	4.89, dt (8.7, 2.0)	72.7, CH	
8	4.84, dd (8.2, 1.8)	105.3, CH	C-6, C-9
9	6.42, dd (8.2, 2.2)	140.5, CH	C-7, C-8, C-11
11	6.82, d (2.1)	138.5, CH	C-12
12		113.9, C	
13	3.22[a]<br>3.92, d (17.8)	34.3, CH_2	C-12, C-14
14		72.4, C	
1′		122.4, C	
2′	7.45, d (2.0)	118.0, CH	C-3′, C-4′, C-6′, C-7′
3′		145.7, C	
4′		153.5, C	
OCH_3-4′	3.92, s	56.0, CH_3	C-4′
5′	7.18, d (8.5)	112.7, CH	C-1′, C-3′
6′	7.50, dd (8.5, 2.0)	124.4, CH	C-2′, C-4′
7′		165.6, C	
1″		123.3, C	
2″	7.70, d (2.2)	126.2, CH	C-3″, C-4″, C-6″
3″		143.0, C	
4″		148.6, C	
OH-4″	9.44, s		
5″	6.85, d (8.2)	116.1, CH	C-1″, C-3″
6″	7.14, dd (8.2, 2.2)	128.2, CH	C-2″, C-4″
7″	3.58, d (13.6)<br>3.85, d (13.6)	35.3, CH_2	C-3, C-4, C-1″, C-2″, C-6″

Supplementary Figures

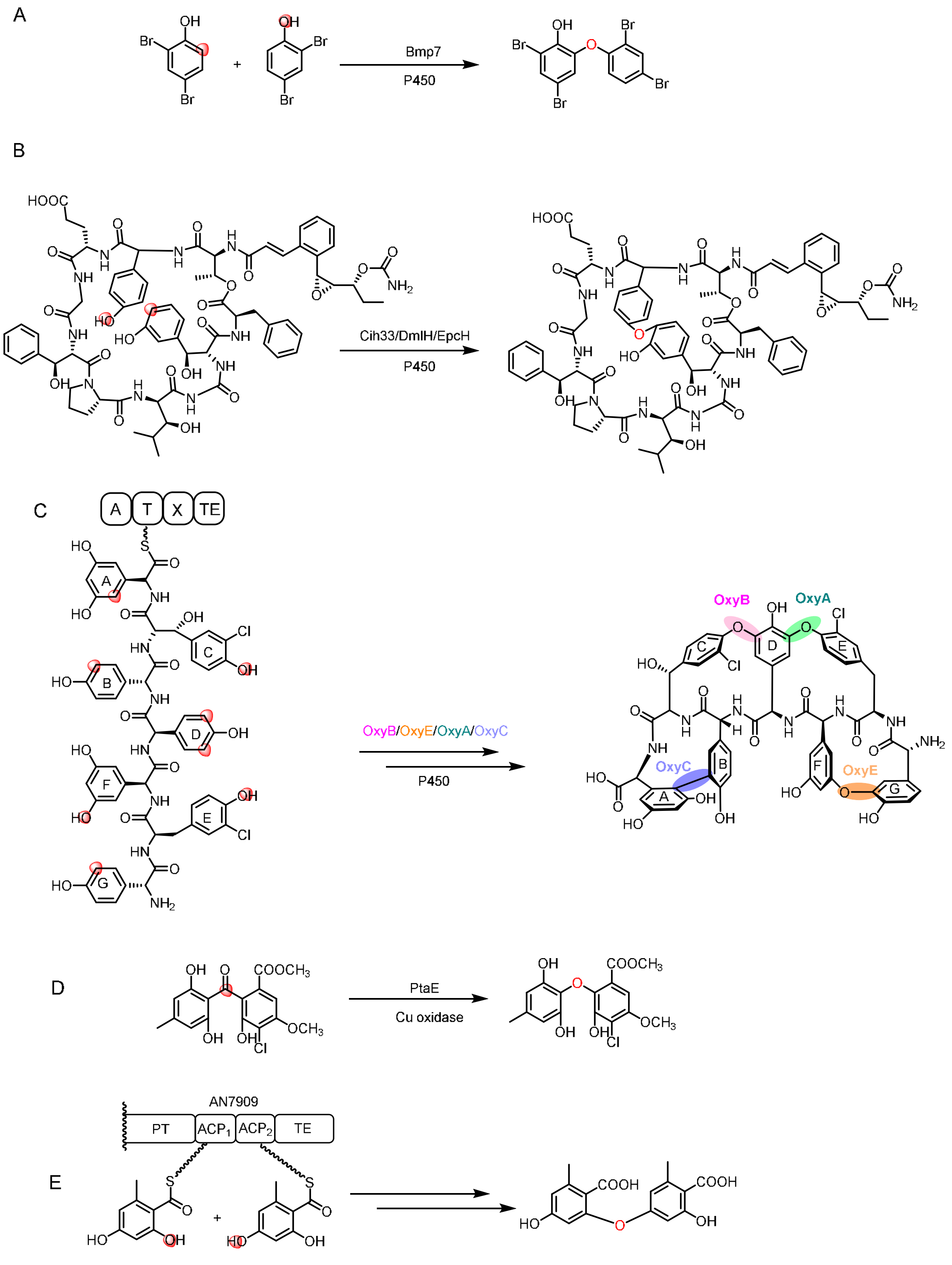

Figure S1. Aryl-aryl ether formation in the biosynthesis of natural sources.

P450-catalyzed aryl-aryl ether formation *via* diphenol coupling by Bmp7 (A), Cih33/DmlH/EpcH (B), OxyB, OxyE, and OxyA (C). Cu oxidase-catalyzed oxidative rearrangement of pestheic acid by PtaE (D). Non-reducing PKS-catalyzed aryl–aryl ether formation (E).

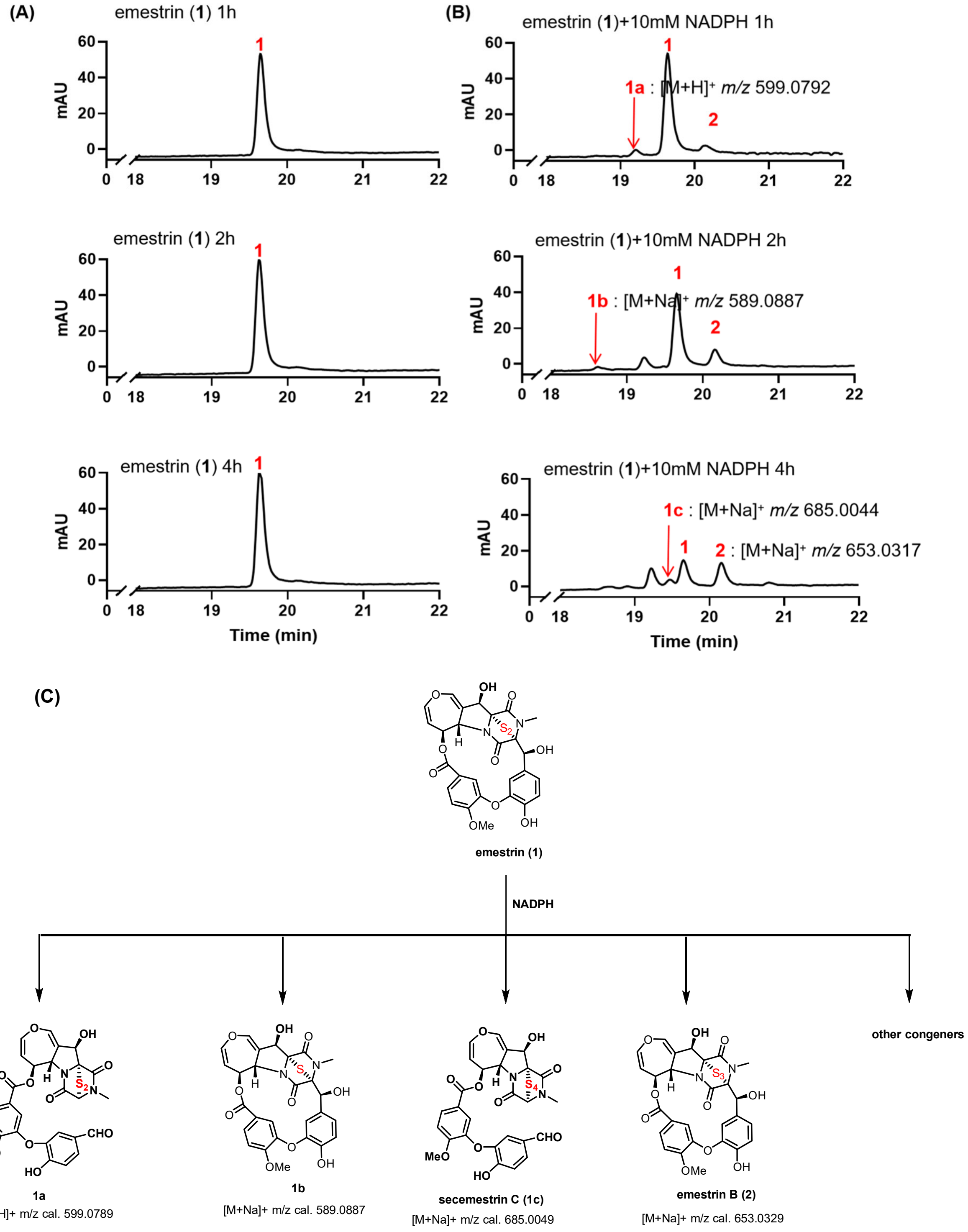

Figure S2. LC-MS profiles of emestrin (**1**) conversion to emestrin B (**2**) in the presence of NADPH.

(A) Emestrin (**1**) in DMSO/H_2O (1:1000) without NADPH. (B) Emestrin (**1**) in DMSO/H_2O (1:1000) with 10 mM NADPH. The mixtures were incubated at 25℃ for 1 h, 2 h, and 4 h. (C) The predicted products of **1a, 1b,** and **1c** were not confirmed by NMR. The absorptions at 254 nm are illustrated.

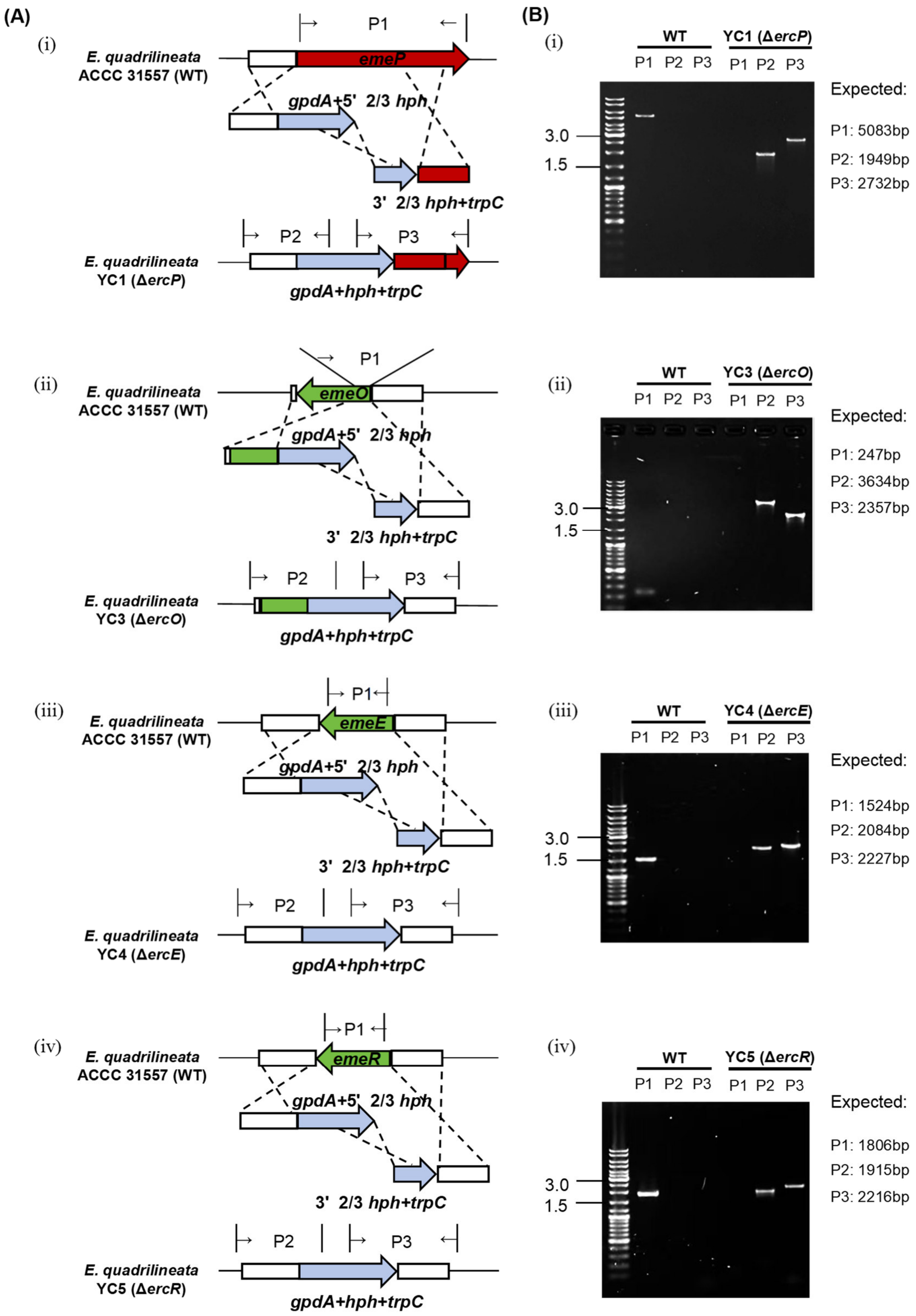

Figure S3. Target gene deletion and PCR verification for *E. quadrilineata* strains.

(A) The split-marker strategy was applied for deletion of *emeP*, *emeO*, *emeE* and *emeR* (Ai–Aiv) and PCR was carried out for verification of *E. quadrilineata* YC1 (Δ*emeP*), YC3 (Δ*emeO*), YC4 (Δ*emeE*), and YC5 (Δ*emeR*) by amplification of different partial fragments (P1-P3) from genomic DNA (Bi–Biv). The primers used for PCR are listed in Table S3.

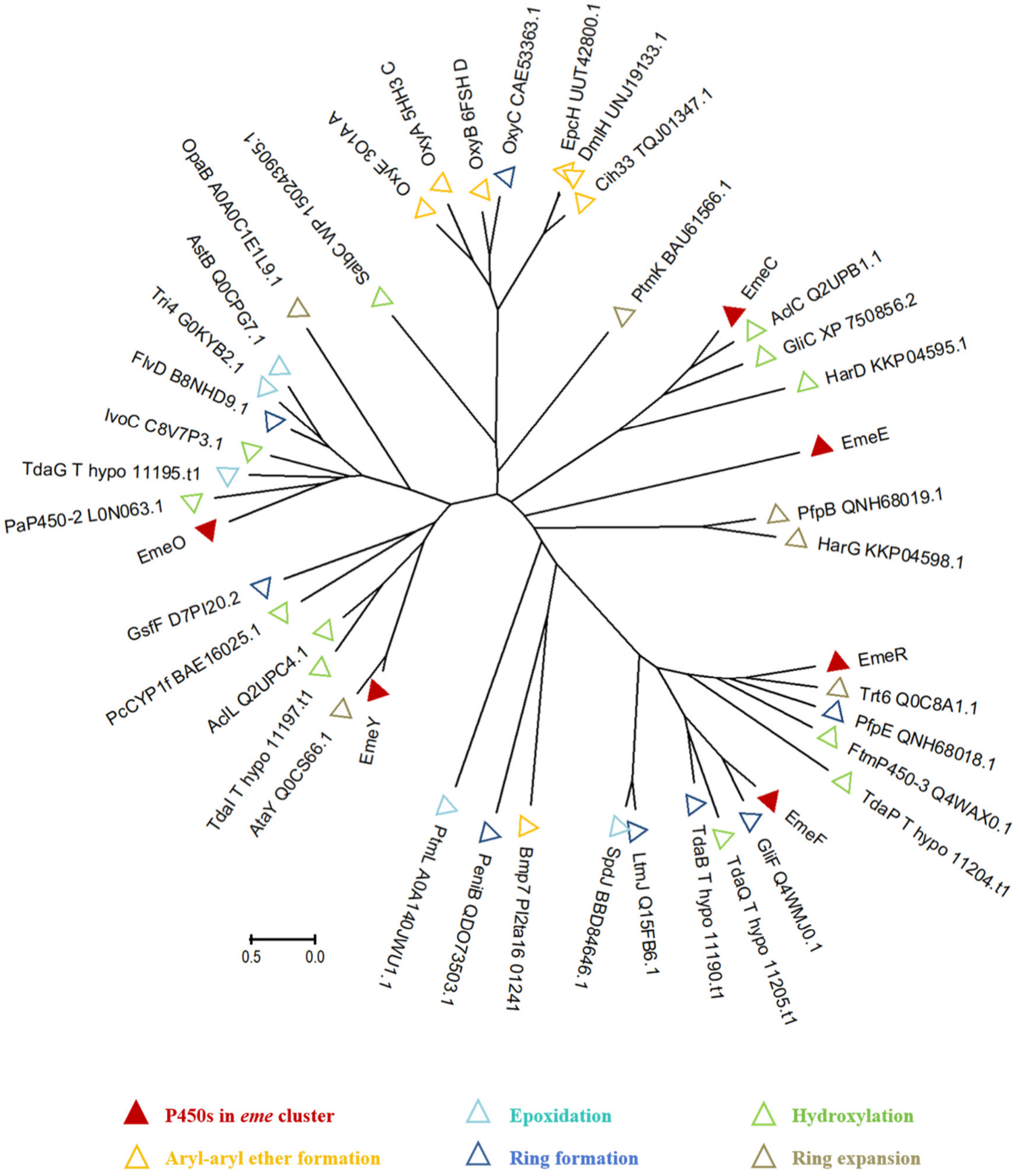

Figure S4. Phylogenetic tree of 44 cytochrome P450 proteins.

Ancestral states were inferred using the Maximum Likelihood method[14] and JTT matrix-based model.[15] The tree with the highest log likelihood (-49313.08) is shown. Initial tree(s) for the heuristic search were obtained automatically by applying Neighbor-Join and BioNJ algorithms to a matrix of pairwise distances estimated using the JTT model. The topology with superior log likelihood value was , then selected. The tree is drawn to scale, with branch lengths measured in the number of substitutions per site. Evolutionary analysis was conducted in MEGA X.[16]

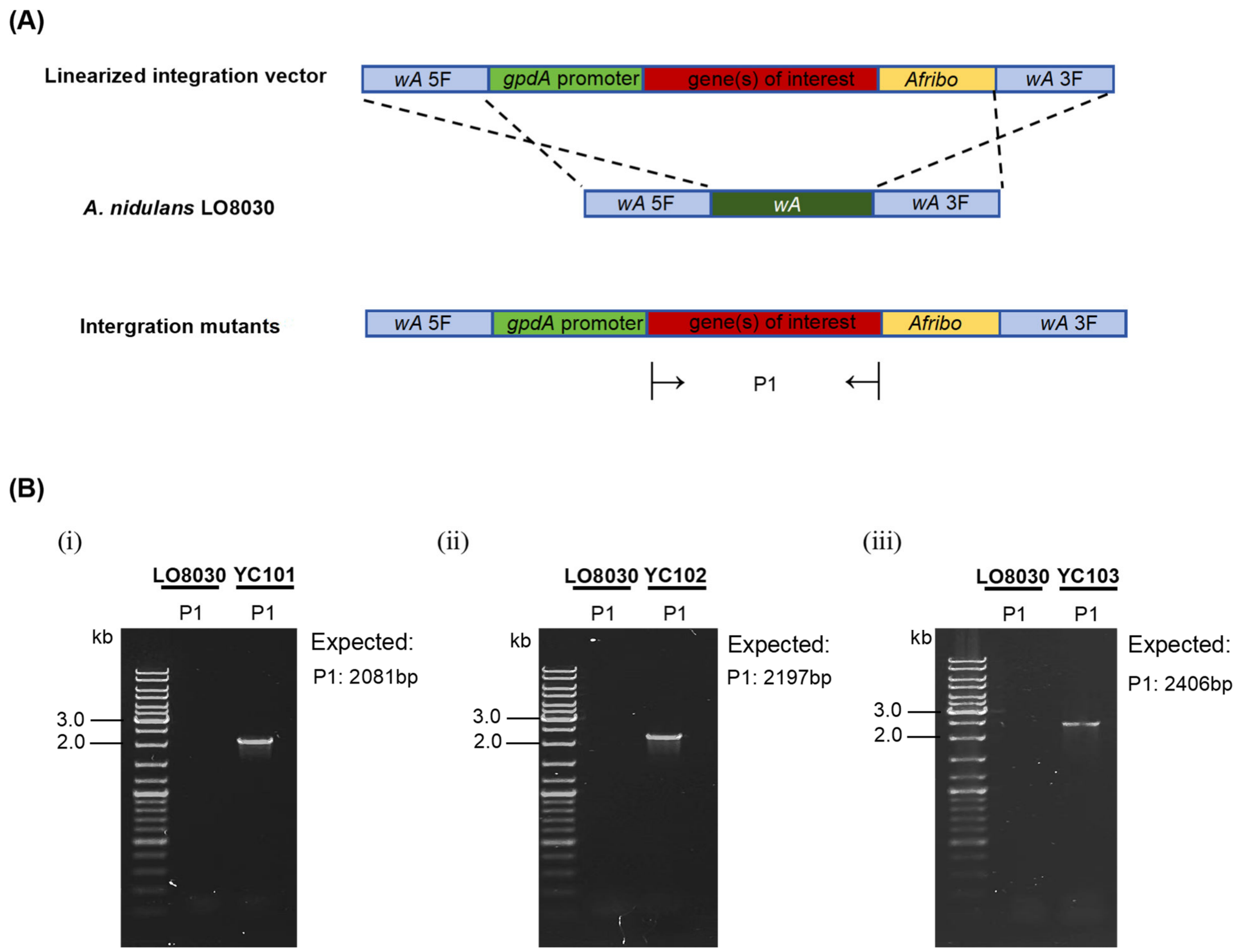

Figure S5. Heterologous expression and PCR verification for *A. nidulans* strains.

(A) Schematic representation of gene integration into the *wA*-PKS locus of *A. nidulans* LO8030. (B) PCR verification for the presence of the target genes *emeO* (i), *emeE* (ii), and *emeR* (iii) was performed with genomic DNA of the *A. nidulans* YC101-103 using primers listed in Table S3.

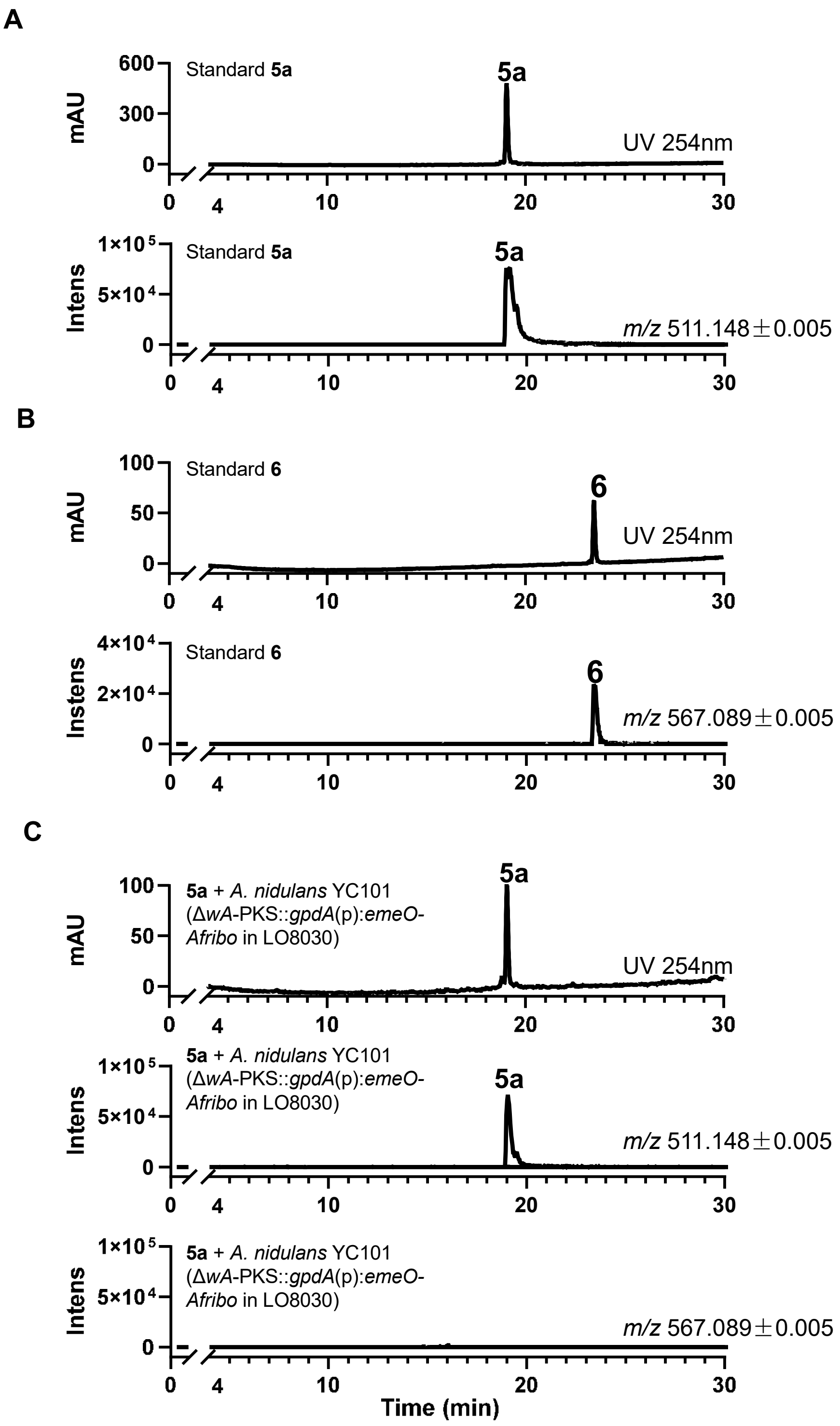

Figure S6. LC-MS analysis of **5a** metabolism by EmeO

LC-MS profiles of the standard **5a** (A) and **6** (B) as well as metabolites from *A. nidulans* YC101 (*emeO* overexpression strian) after feeding with **5a** (C). The chromatograms are illustrated for absorptions at 254 nm. The $[M+Na]^+$ ion of **5a** at *m/z* 511.148 and $[M+H]^+$ ion of **6** at *m/z* 567.089 with a tolerance range of ± 0.005 were also used for detection of substrates and products.

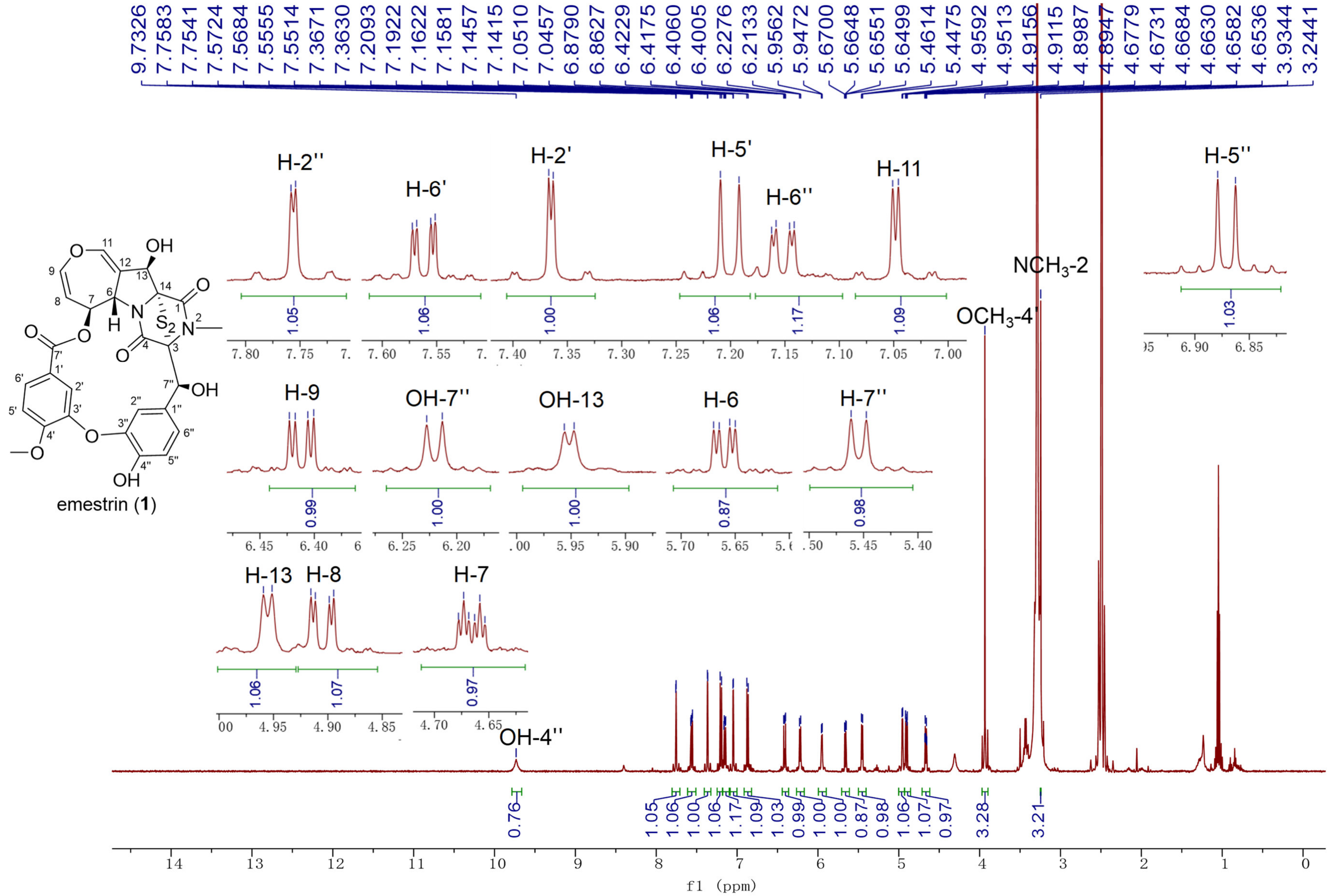

Figure S7. The ^1H NMR spectrum of emestrin (**1**) in DMSO-d_6 (500 MHz).

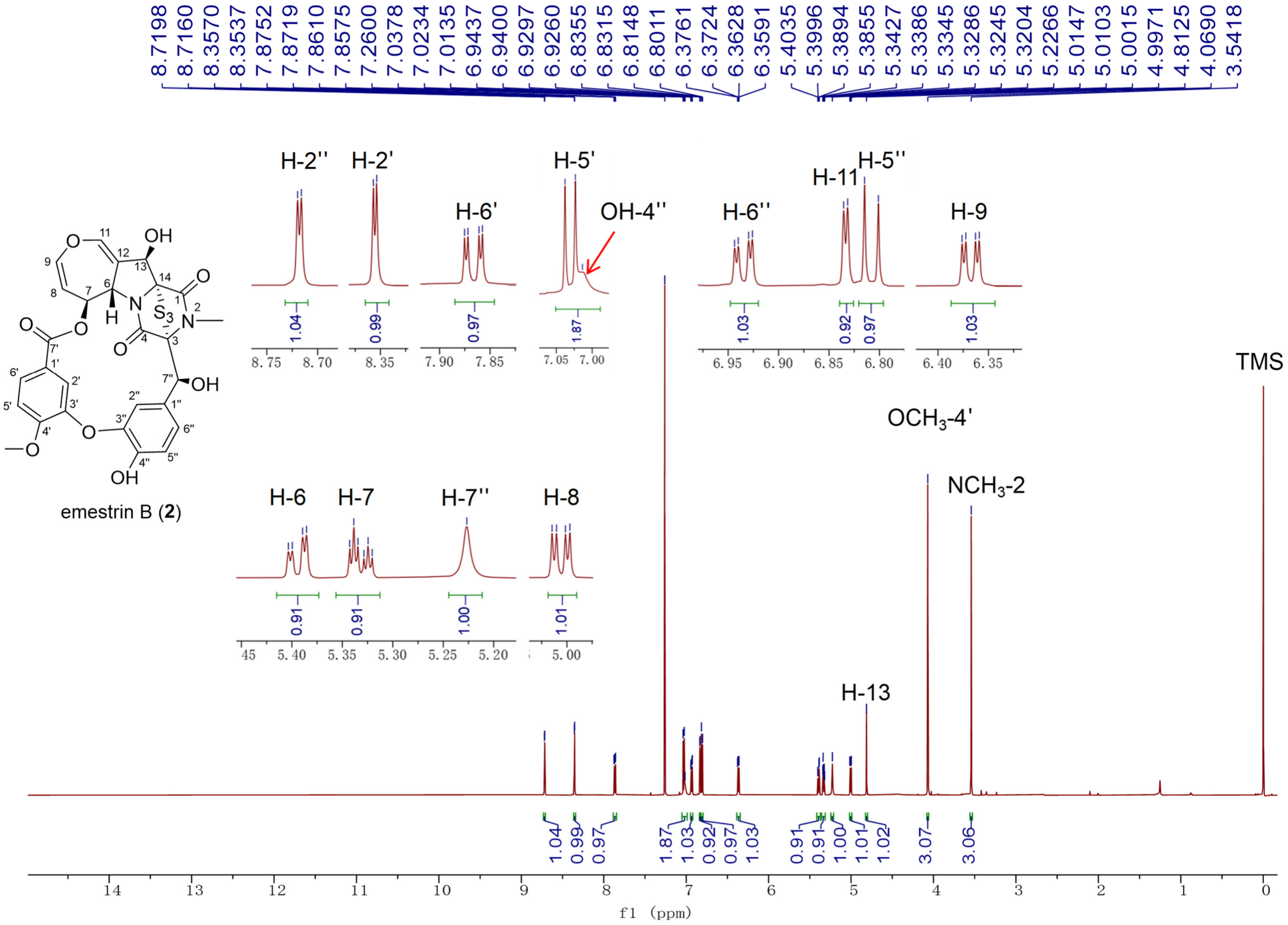

Figure S8. The ^{1}H NMR spectrum of emestrin B (**2**) in $CDCl_3$ (600 MHz).

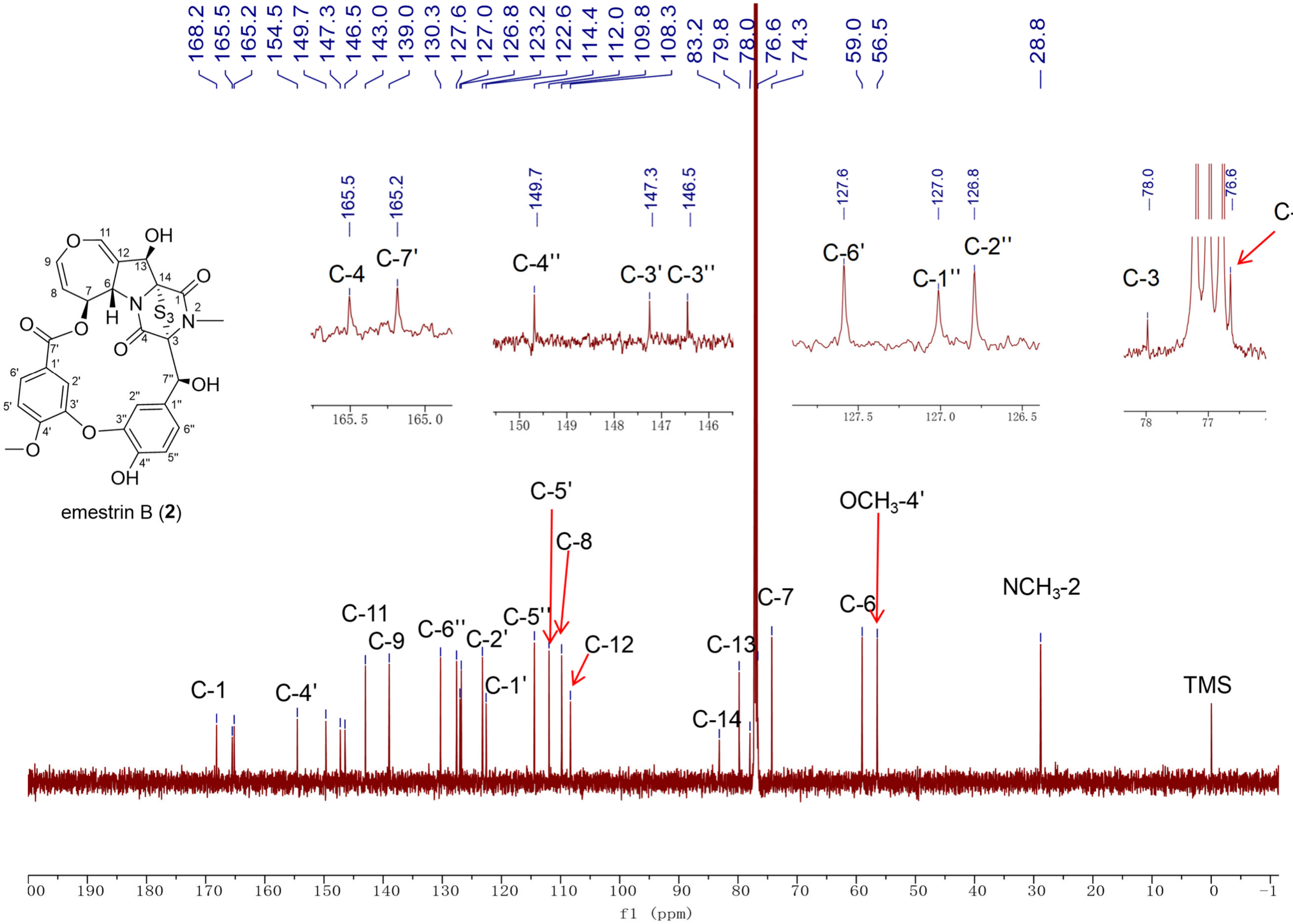

Figure S9. The ^{13}C {^{1}H} NMR spectrum of emestrin B (**2**) in in $CDCl_3$ (150 MHz).

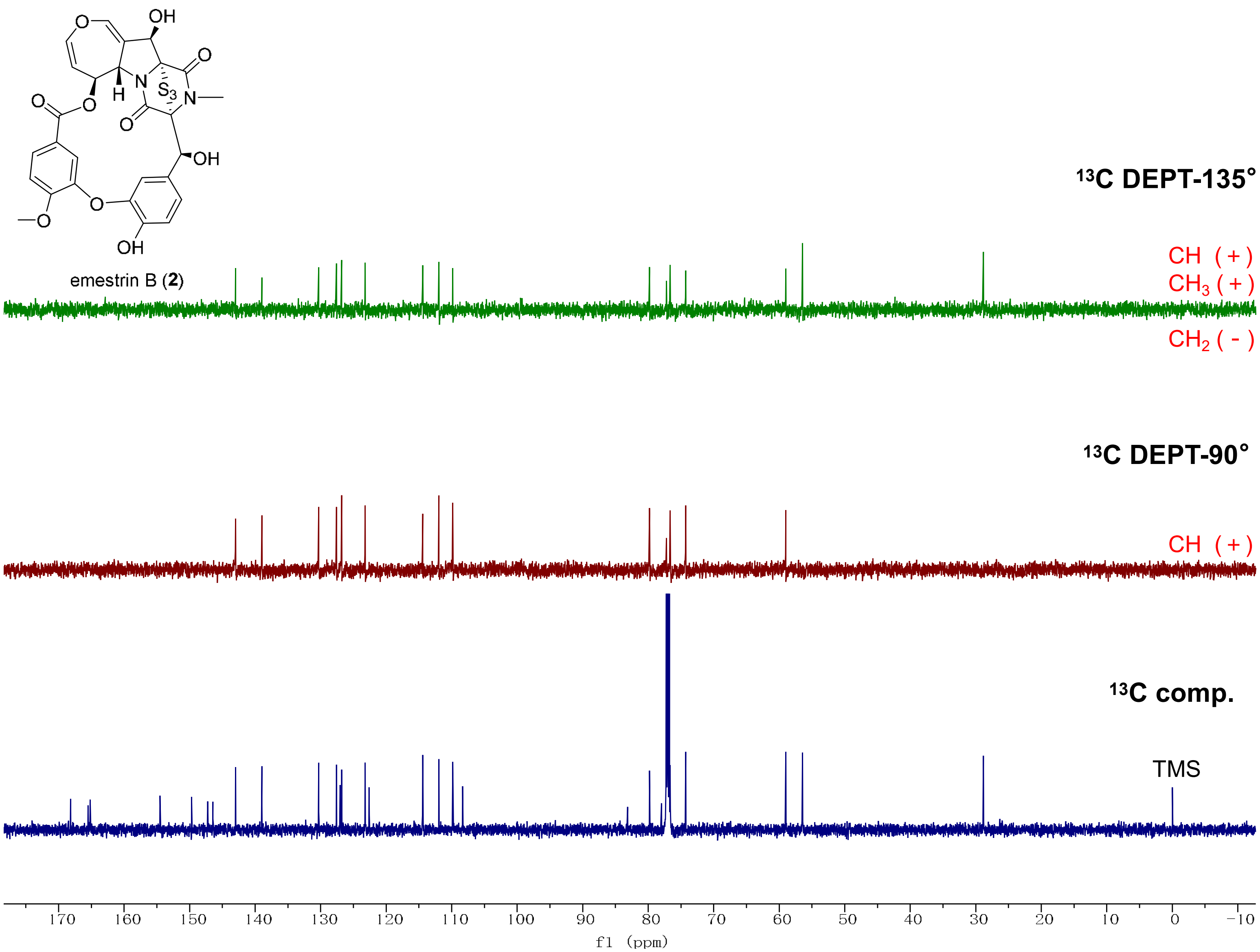

Figure S10. The DEPT spectrum of emestrin B (**2**) in $CDCl_3$ (150 MHz).

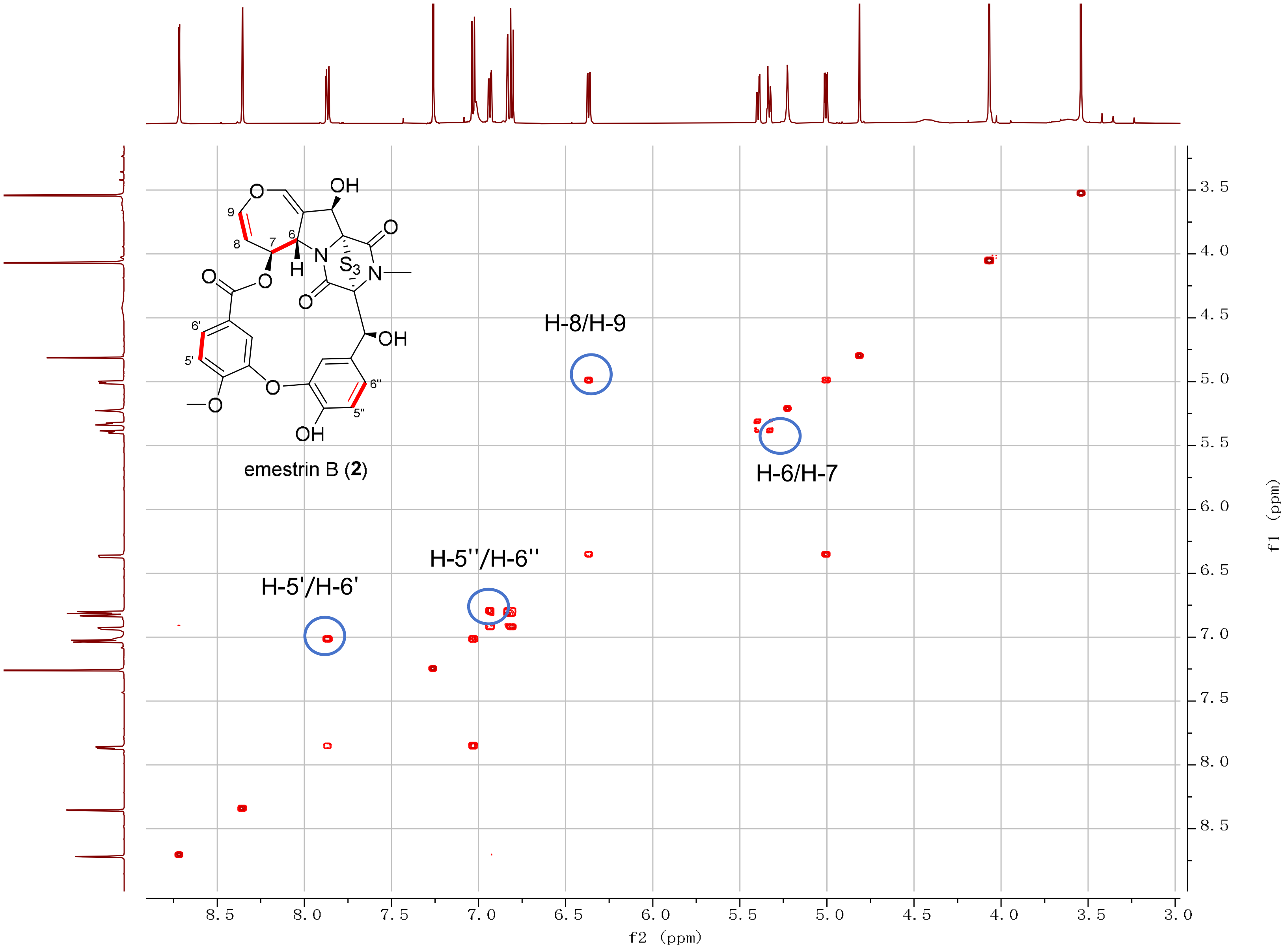

Figure S11. The ^{1}H-^{1}H COSY spectrum of emestrin B (**2**) in $CDCl_3$.

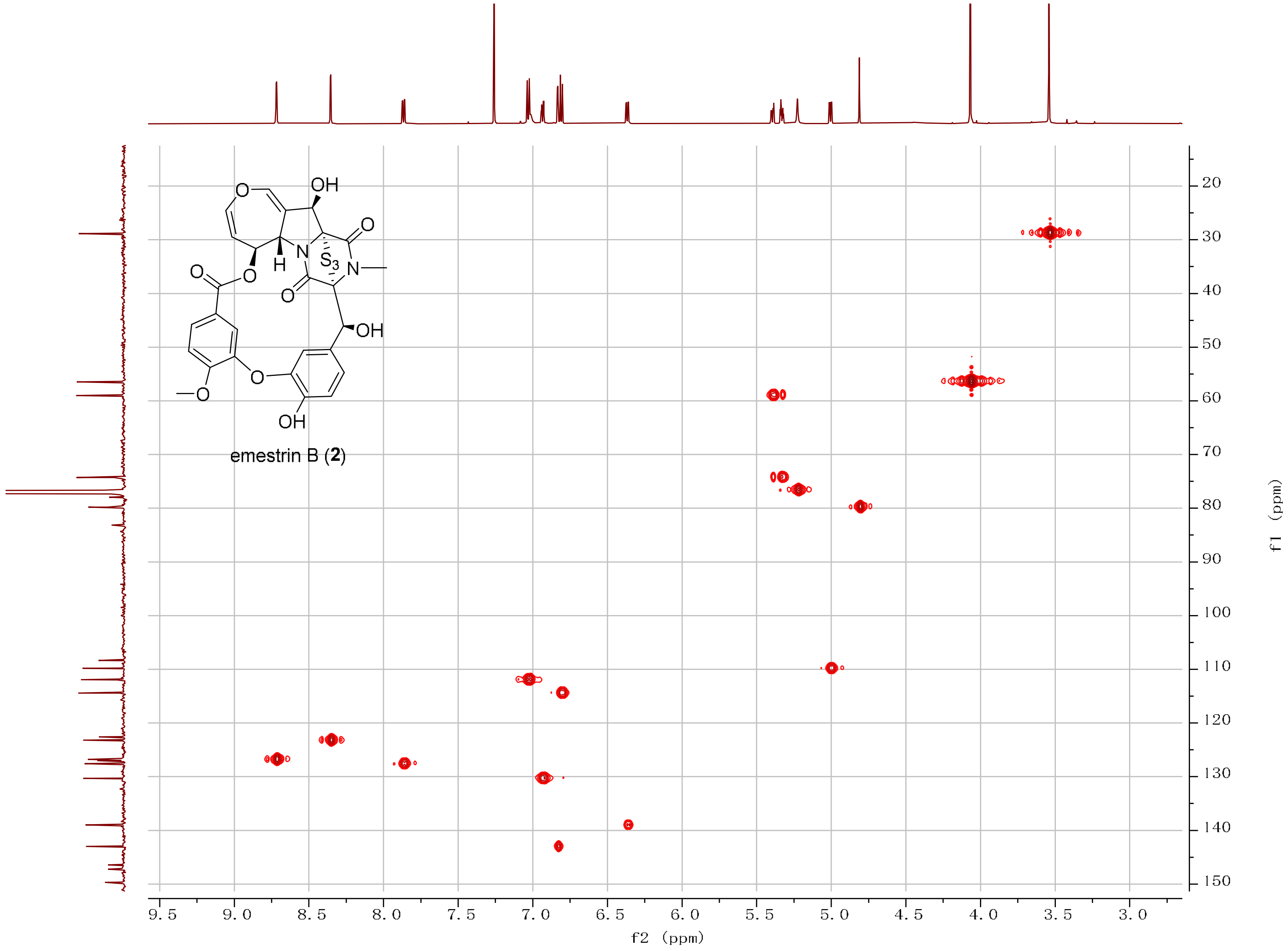

Figure S12. The HSQC spectrum of emestrin B (**2**) in $CDCl_3$.

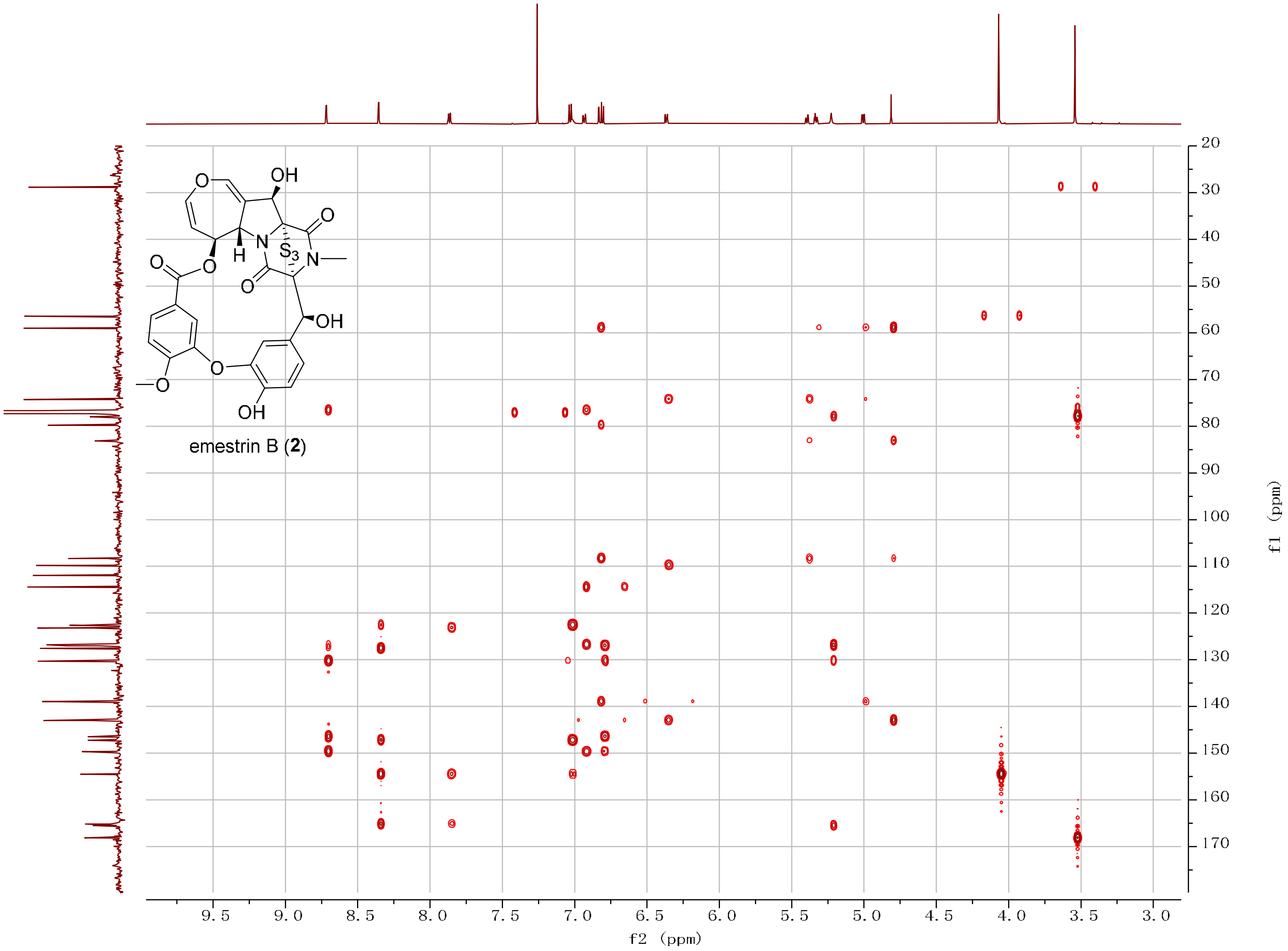

Figure S13. The HMBC spectrum of emestrin B (**2**) in $CDCl_3$.

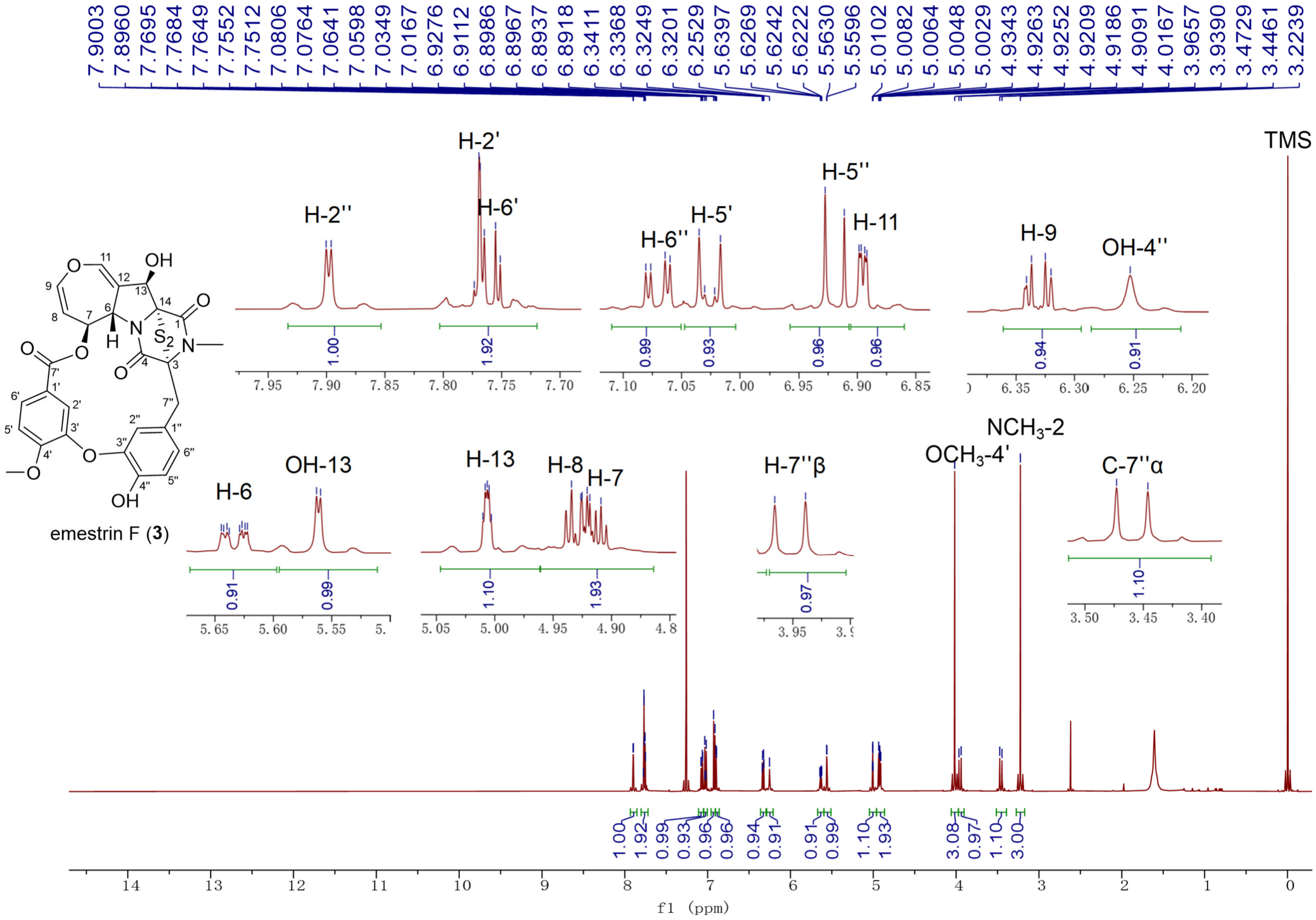

Figure S14. The ^{1}H NMR spectrum of emestrin F (**3**) in $CDCl_3$ (500 MHz).

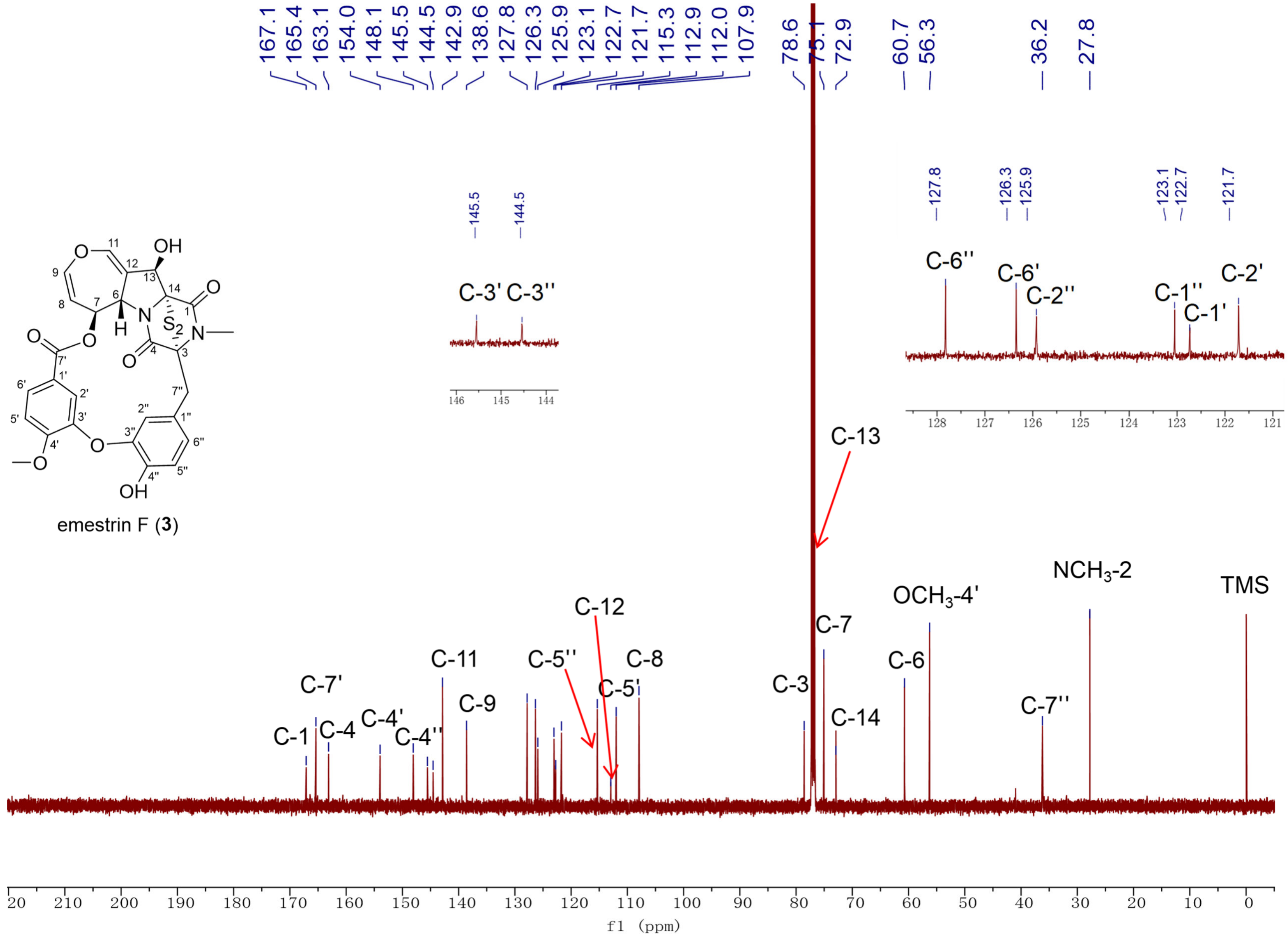

Figure S15. The ^{13}C {^{1}H} NMR spectrum of emestrin F (**3**) in in $CDCl_3$ (125 MHz).

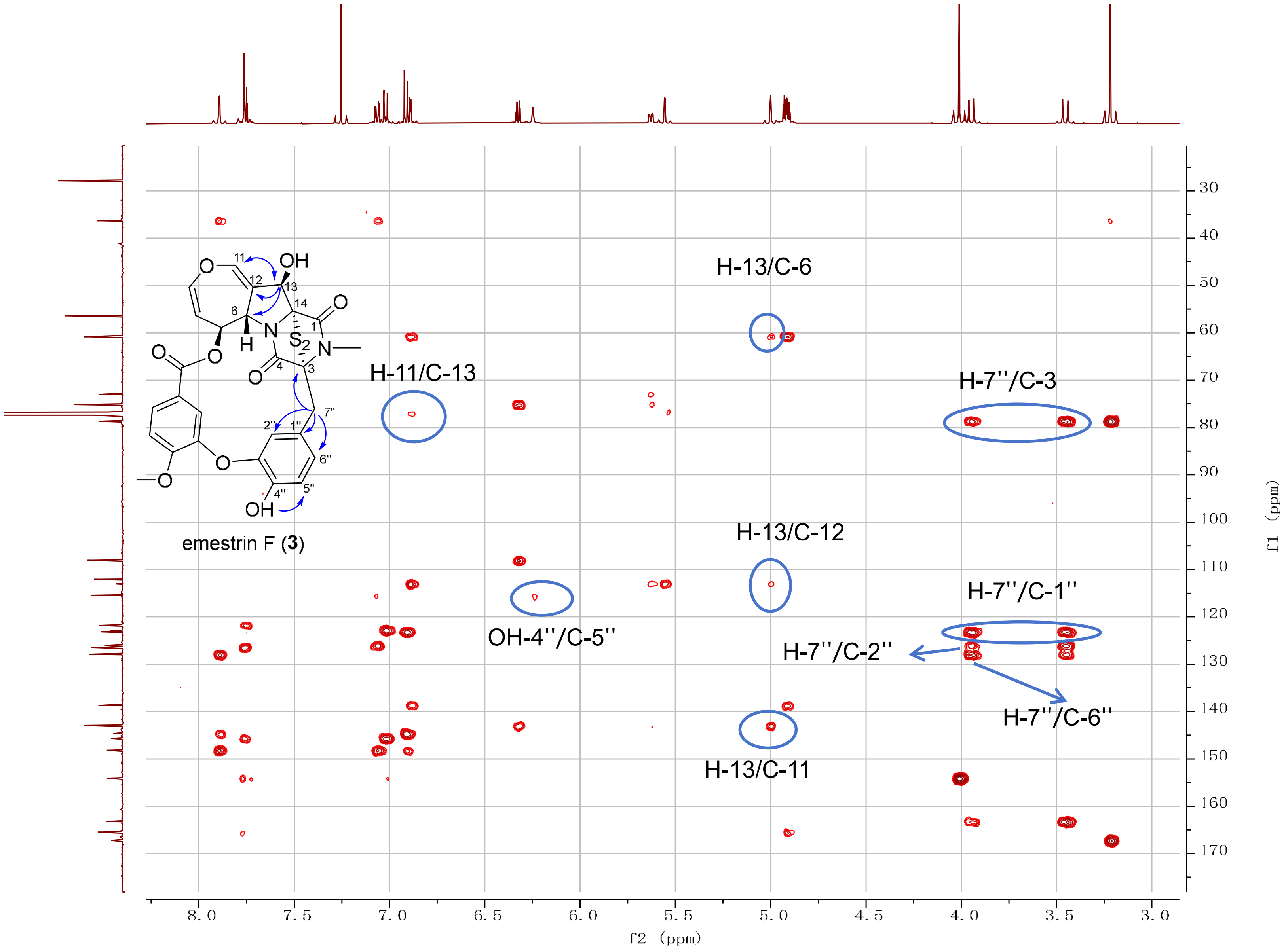

Figure S16. The HMBC spectrum of emestrin F (**3**) in $CDCl_3$.

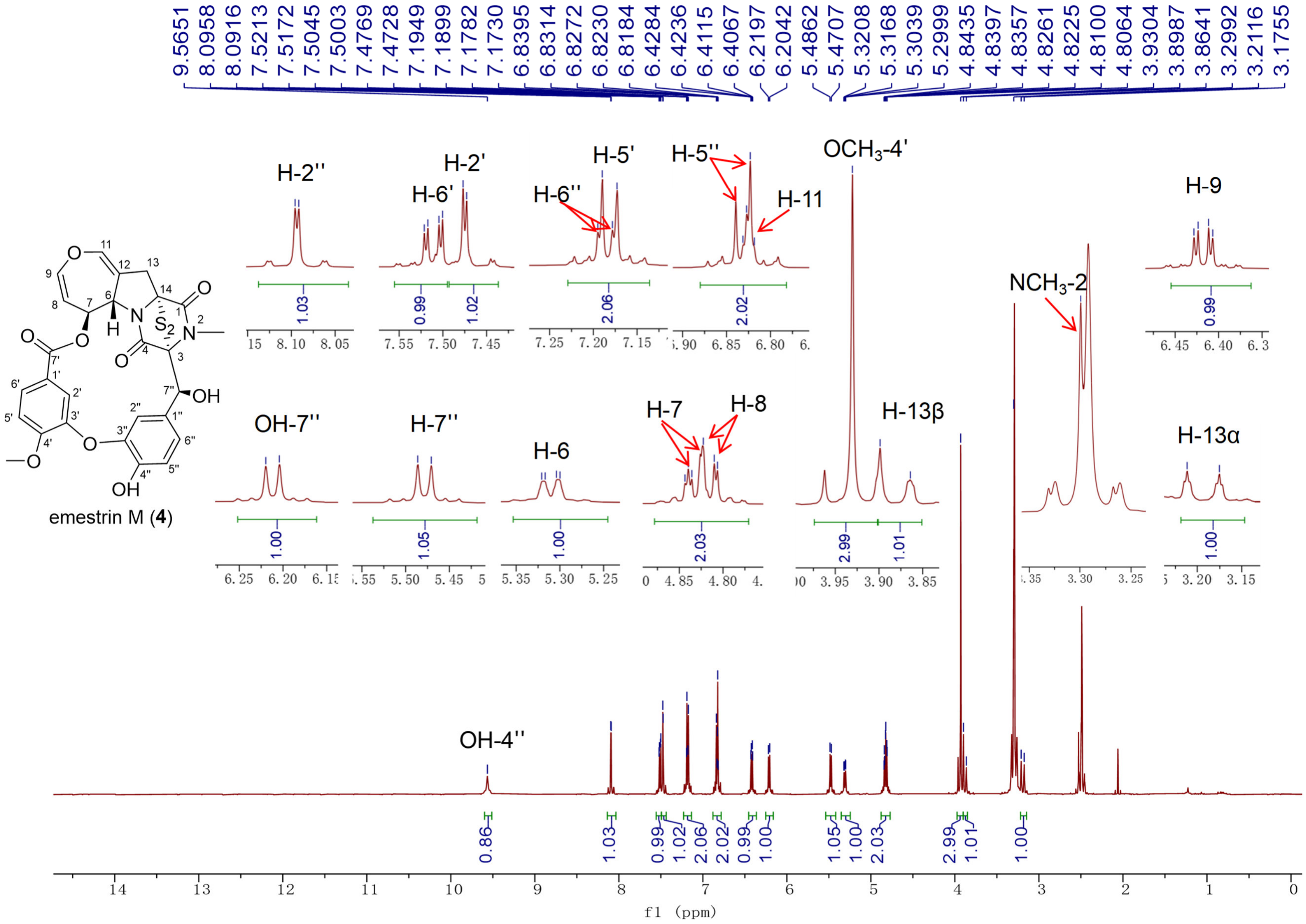

Figure S17. The ^{1}H NMR spectrum of emestrin M (**4**) in DMSO-d_6 (500 MHz).

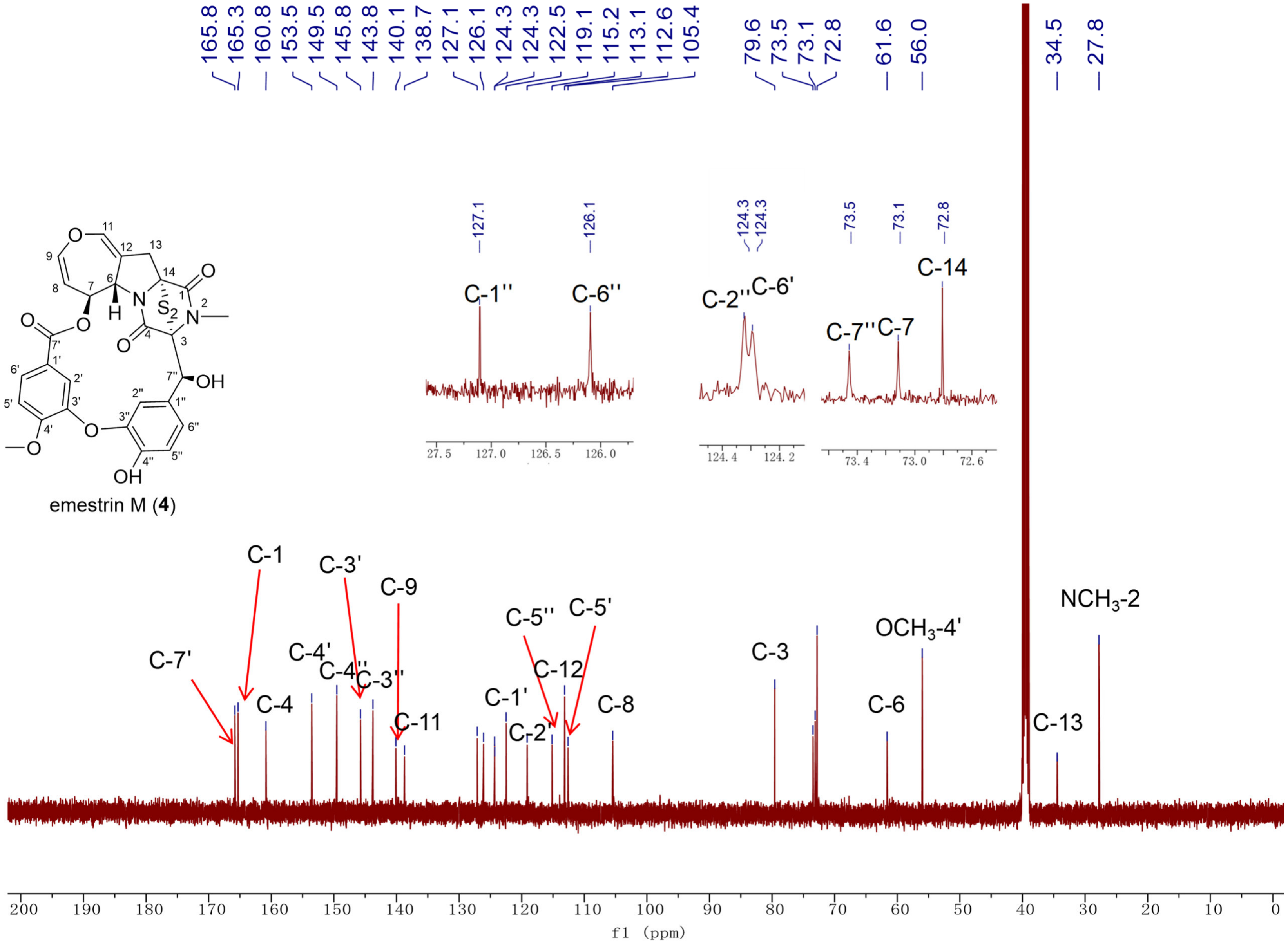

Figure S18. The $^{13}C\ \{^{1}H\}$ NMR spectrum of emestrin M (**4**) in DMSO-d_6 (125 MHz).

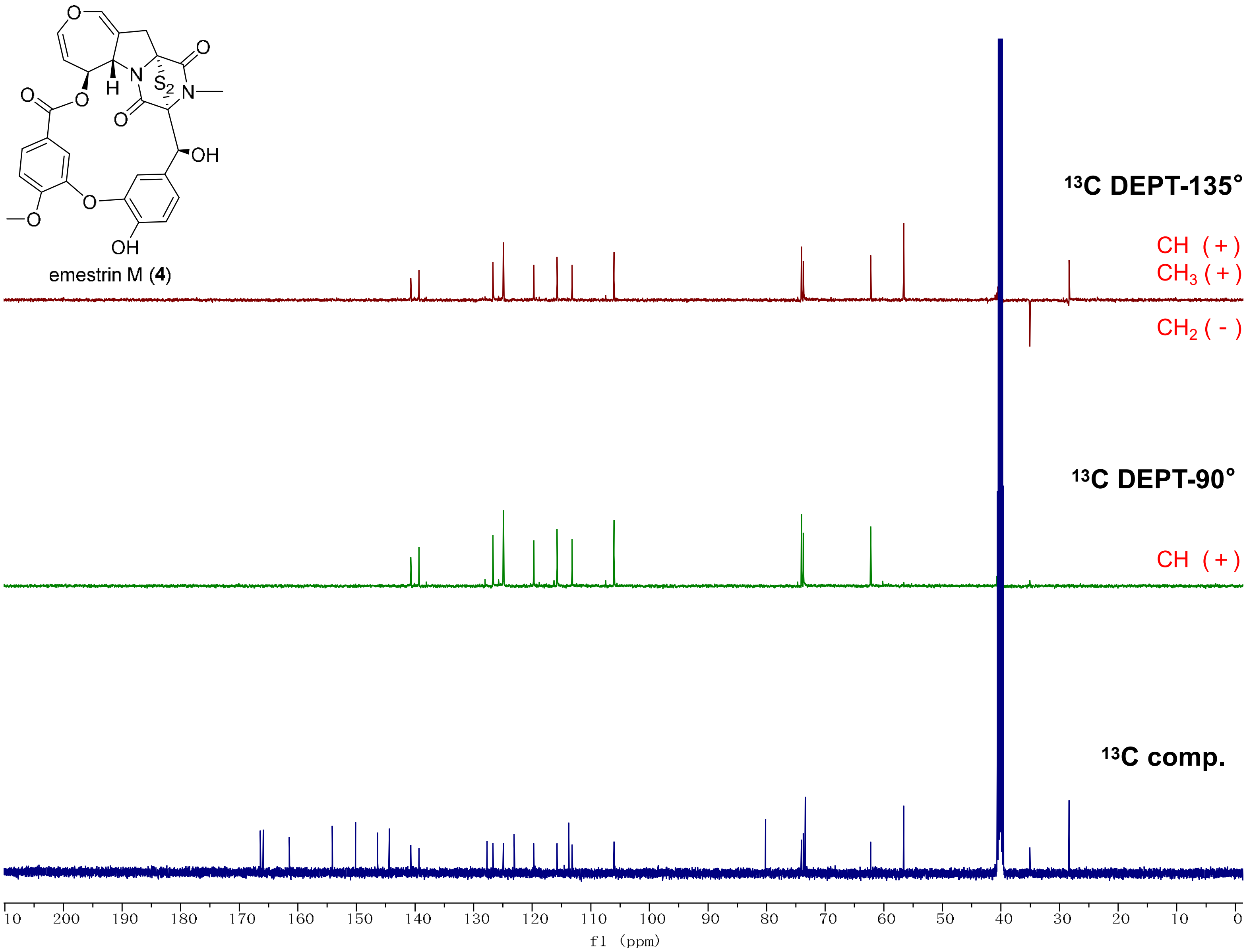

Figure S19. The DEPT spectrum of emestrin M (**4**) in DMSO-d_6 (125 MHz).

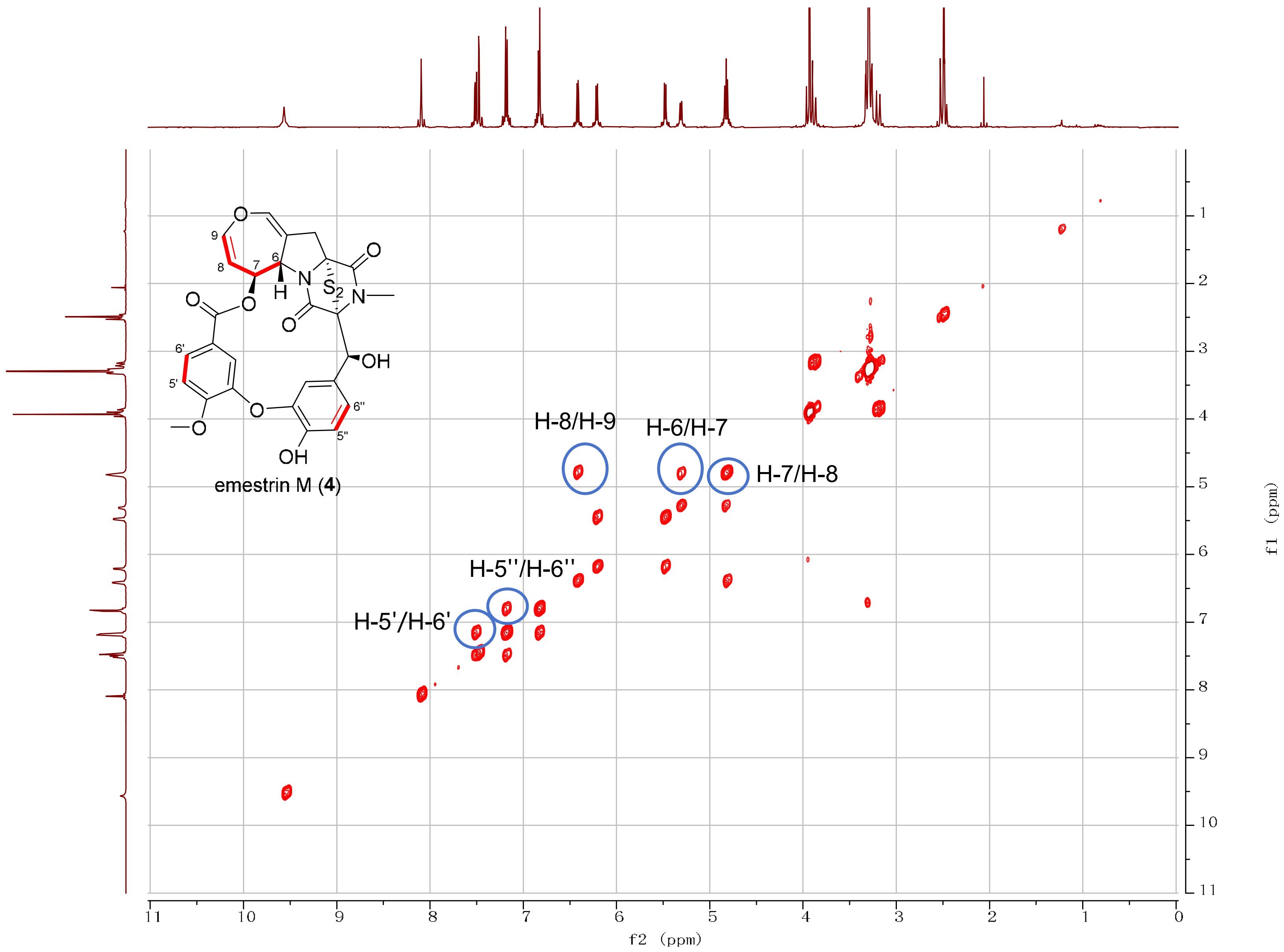

Figure S20. The ^{1}H-^{1}H COSY spectrum of emestrin M (**4**) in DMSO-d_6.

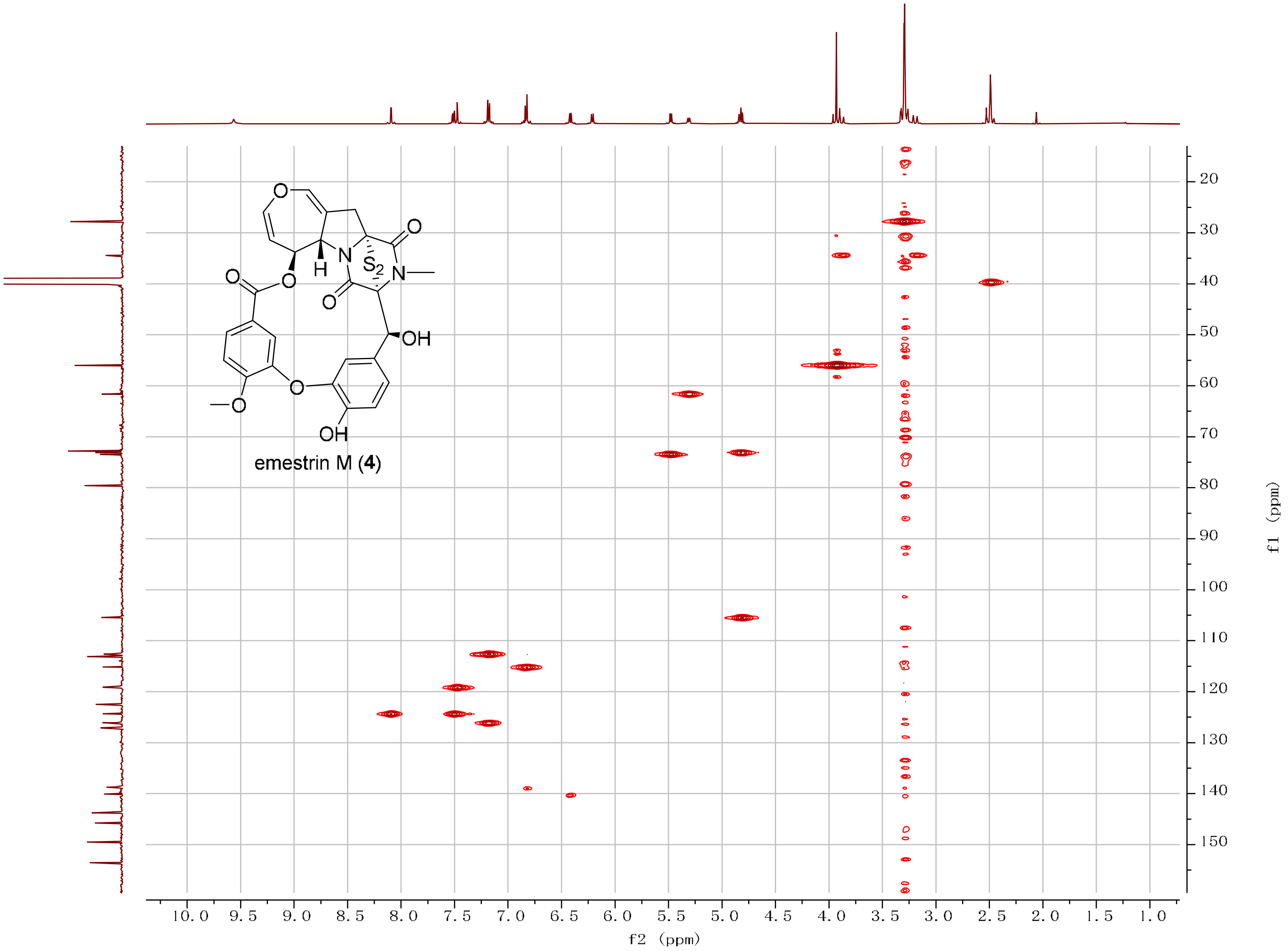

Figure S21. The HSQC spectrum of emestrin M (**4**) in DMSO-d_6.

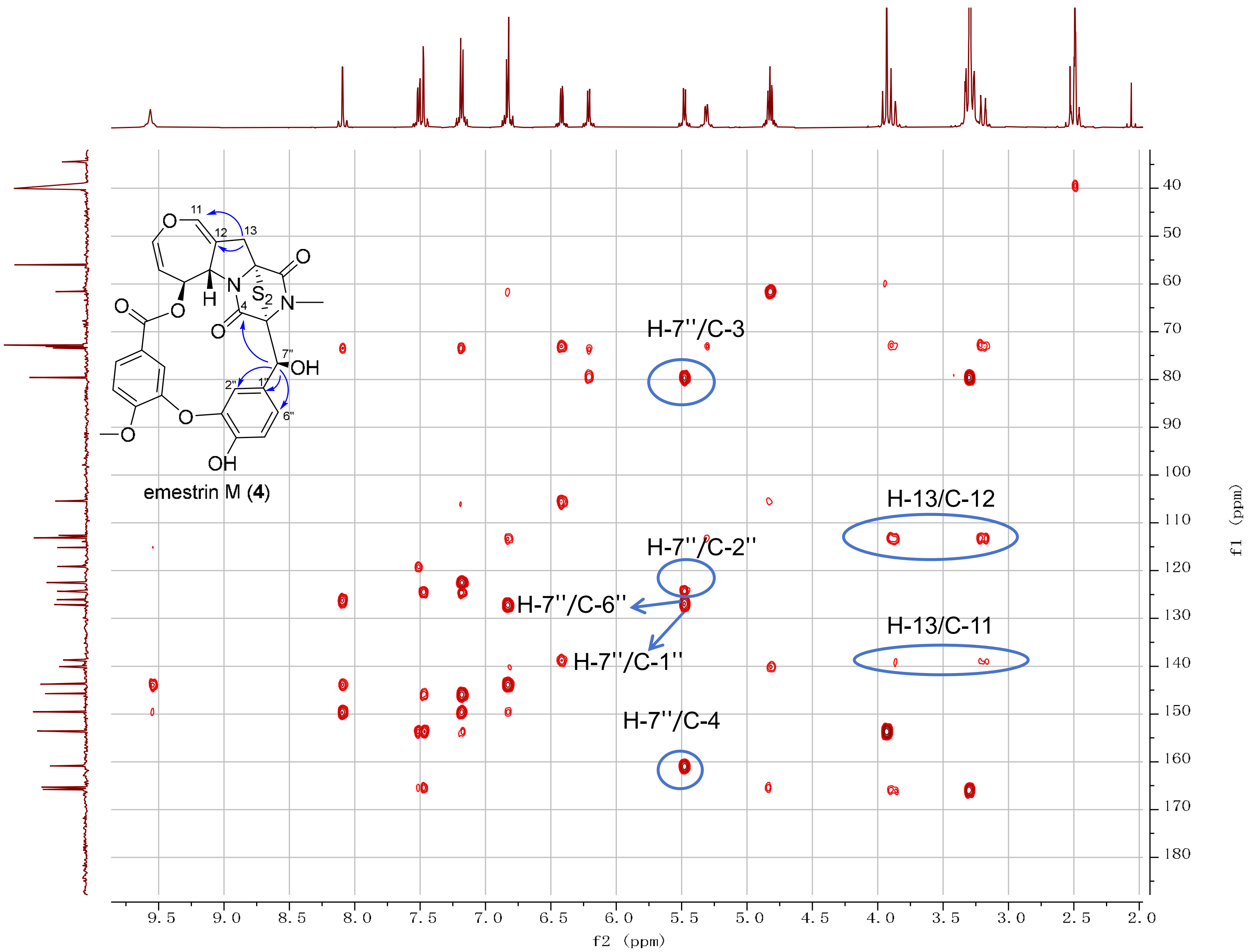

Figure S22. The HMBC spectrum of emestrin M (**4**) in DMSO-d_6.

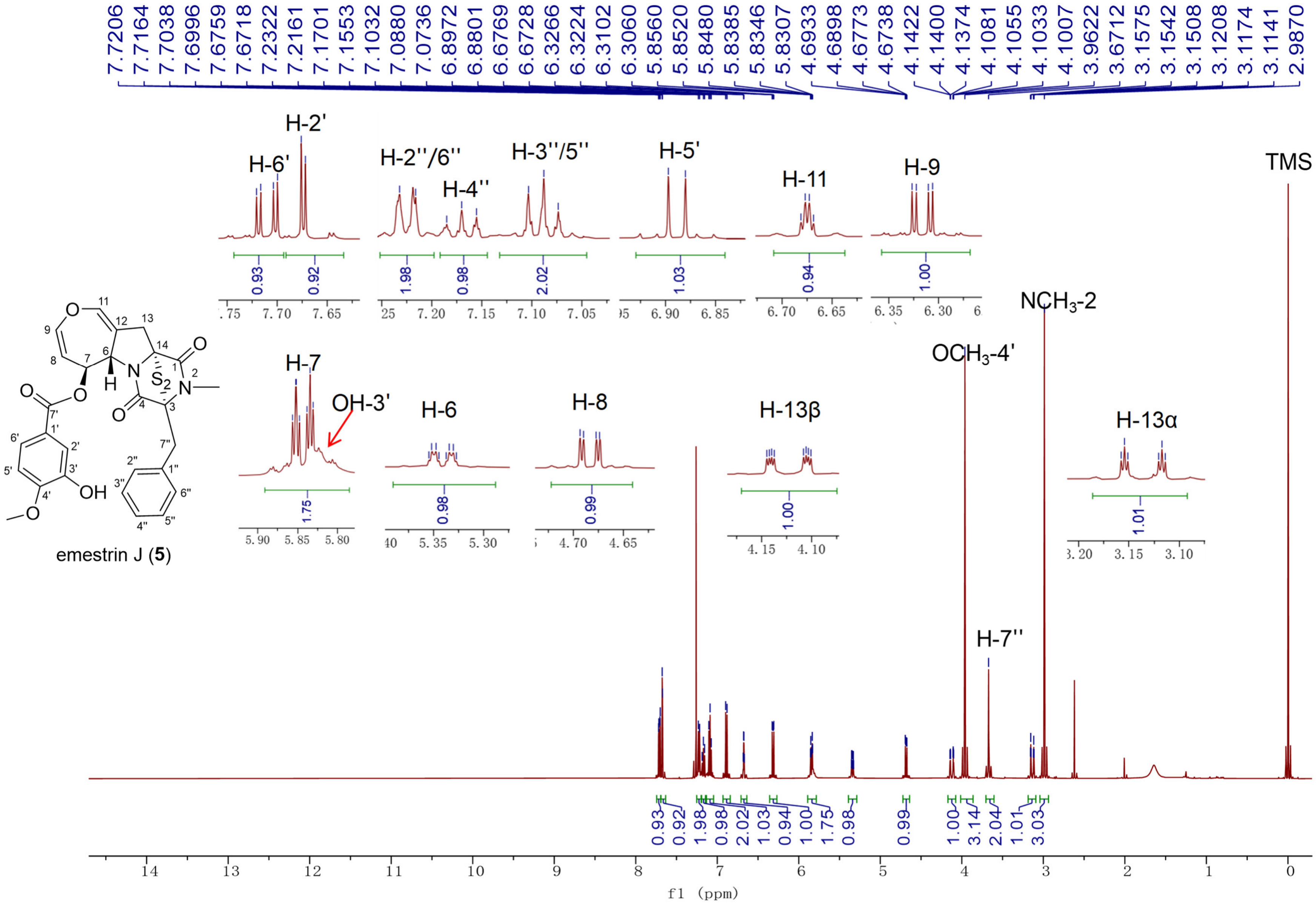

Figure S23. The ^{1}H NMR spectrum of emestrin J (**5**) in $CDCl_3$ (500 MHz).

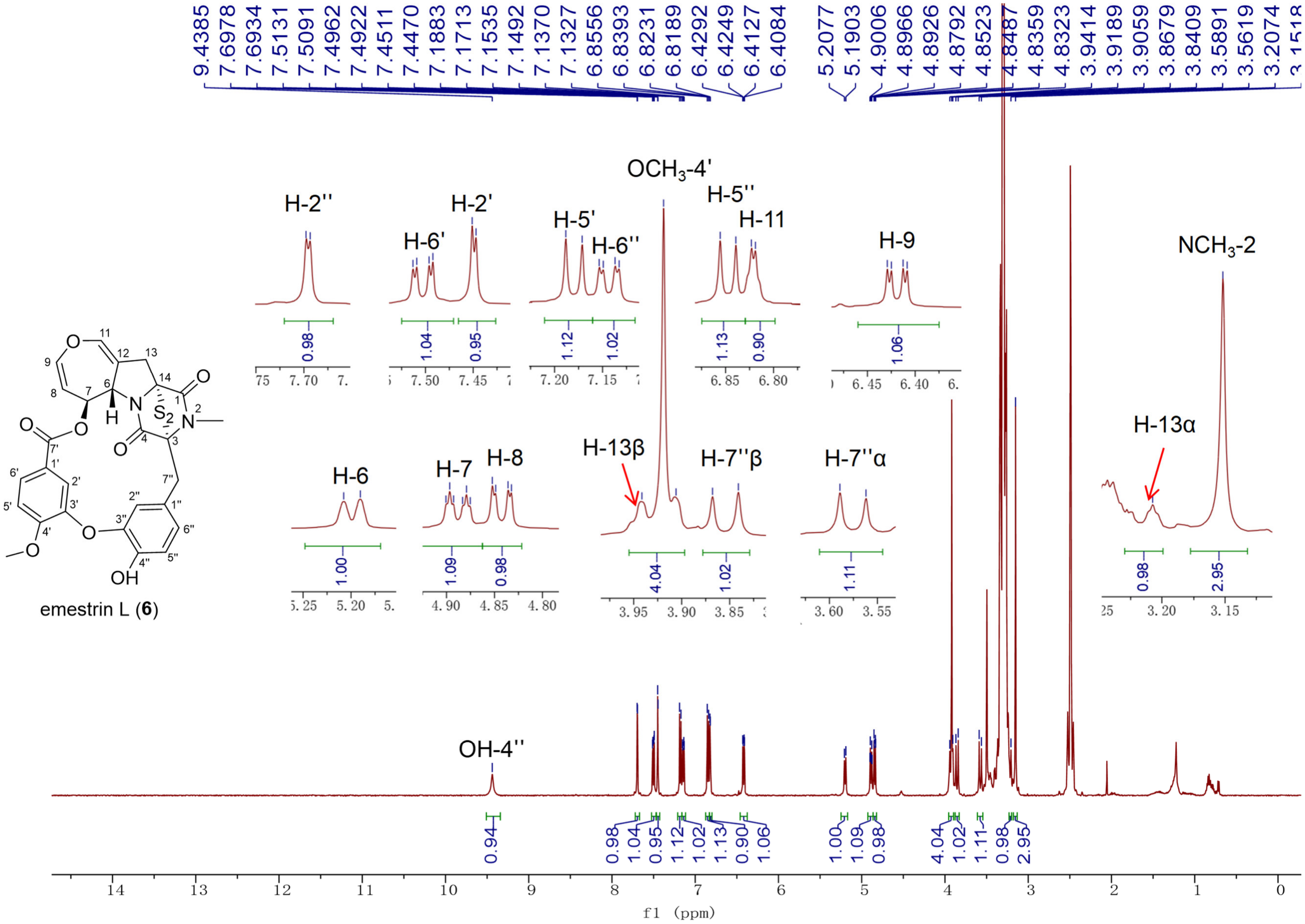

Figure S24. The ^{1}H NMR spectrum of emestrin L (**6**) in DMSO-d_6 (500 MHz).

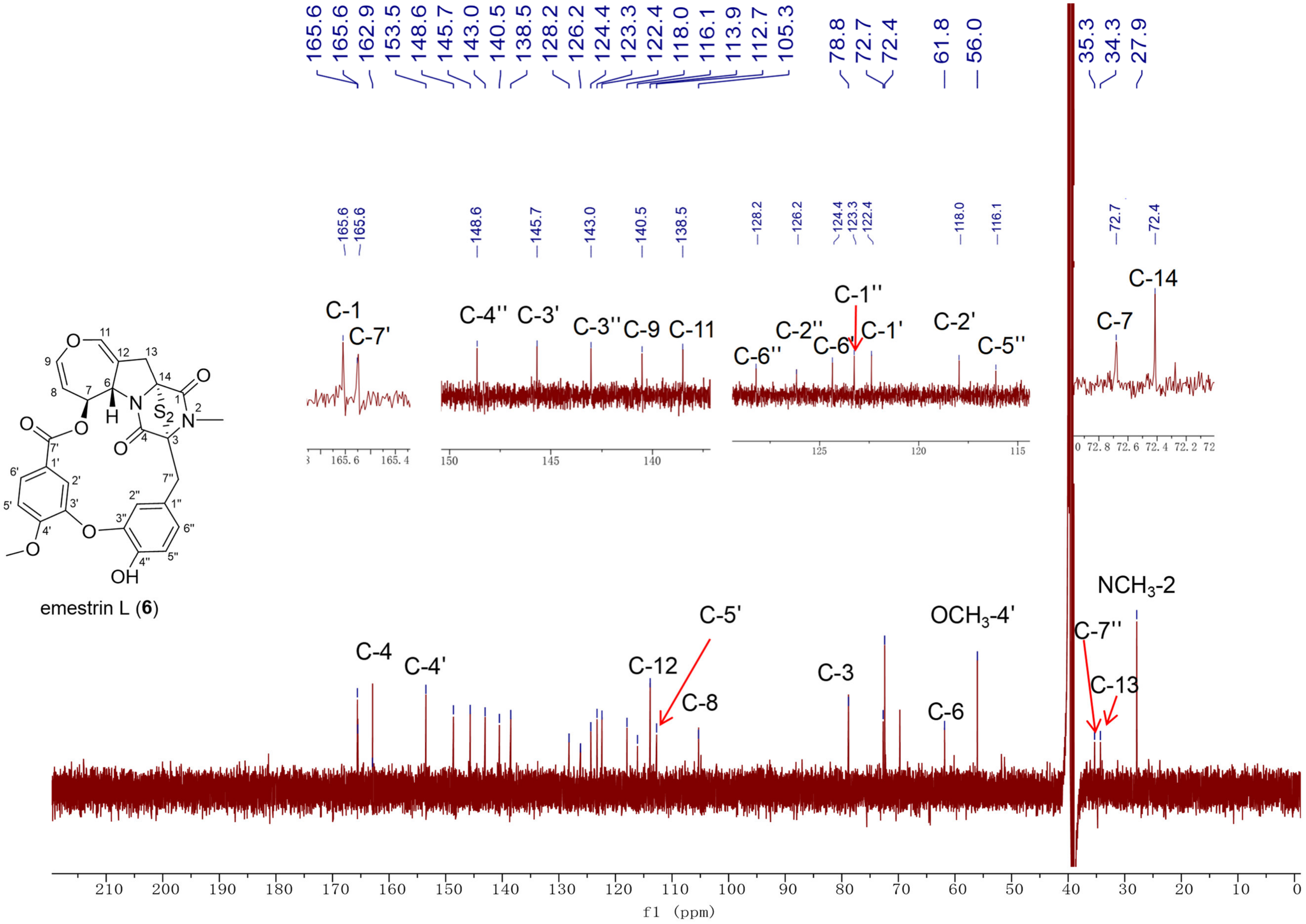

Figure S25. The 13C {1H} NMR spectrum of emestrin L (**6**) in DMSO-d_6 (125 MHz).

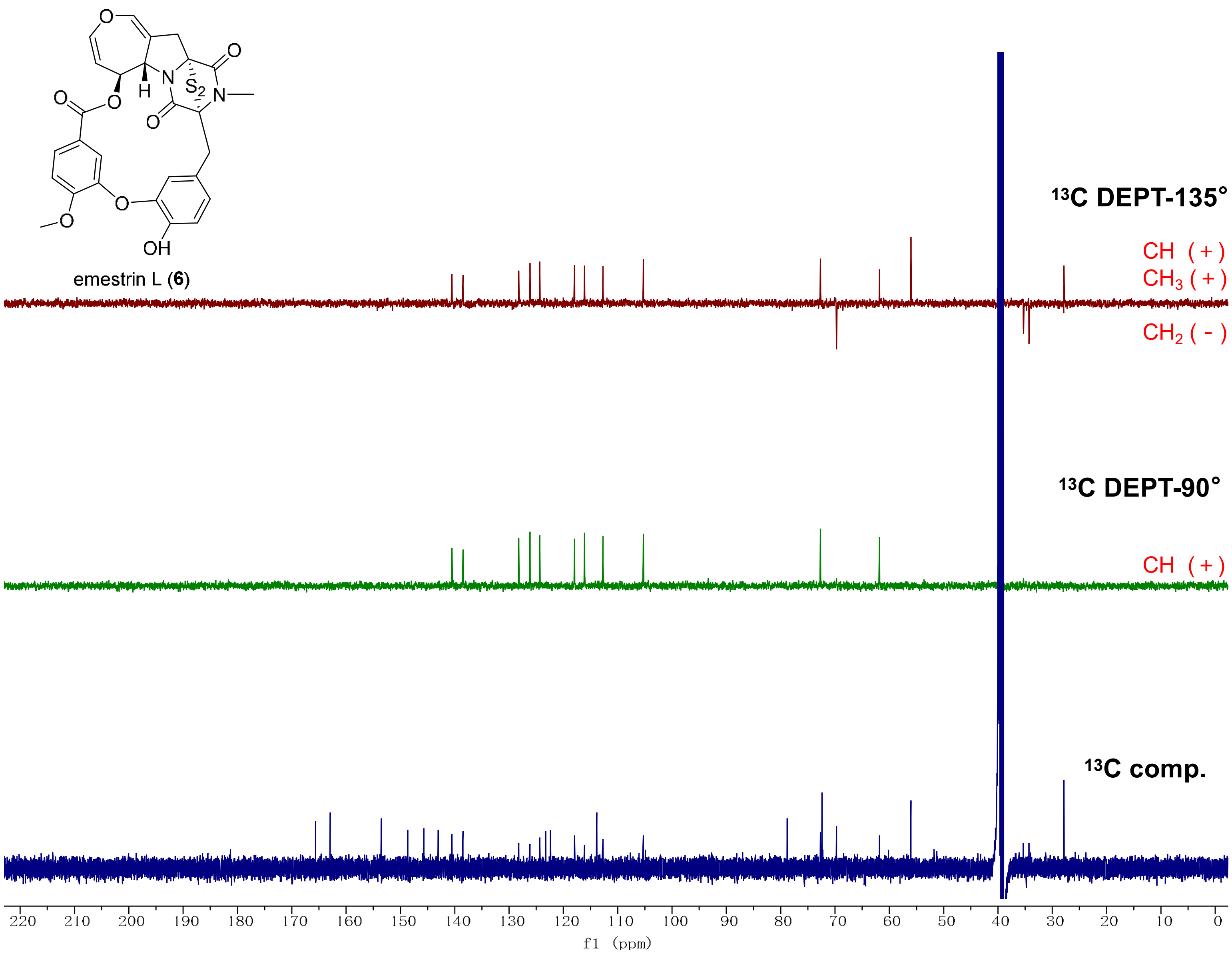

Figure S26. The DEPT spectrum of emestrin L (**6**) in DMSO-d_6 (125 MHz).

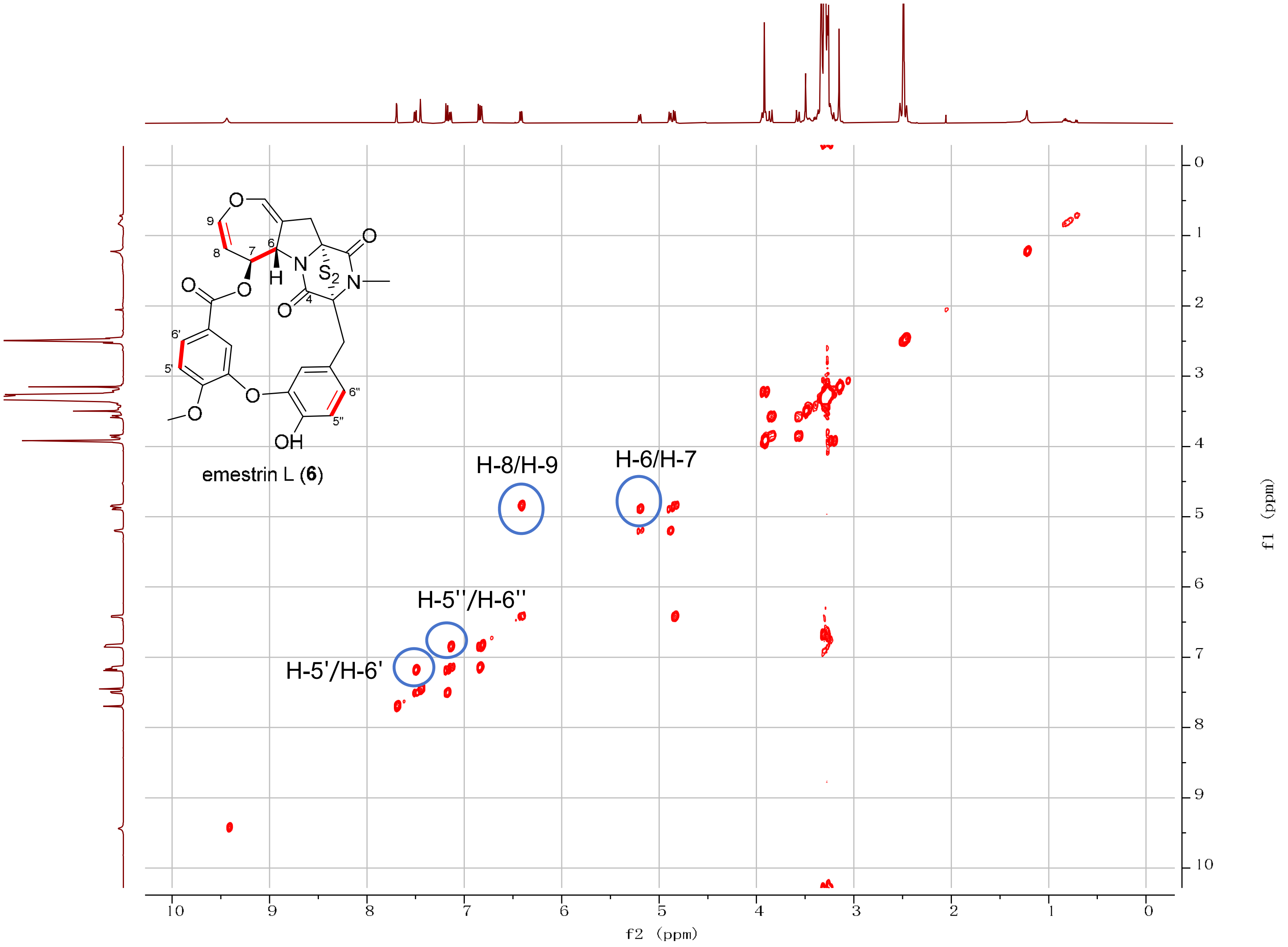

Figure S27. The ^{1}H-^{1}H COSY spectrum of emestrin L (**6**) in DMSO-d_6.

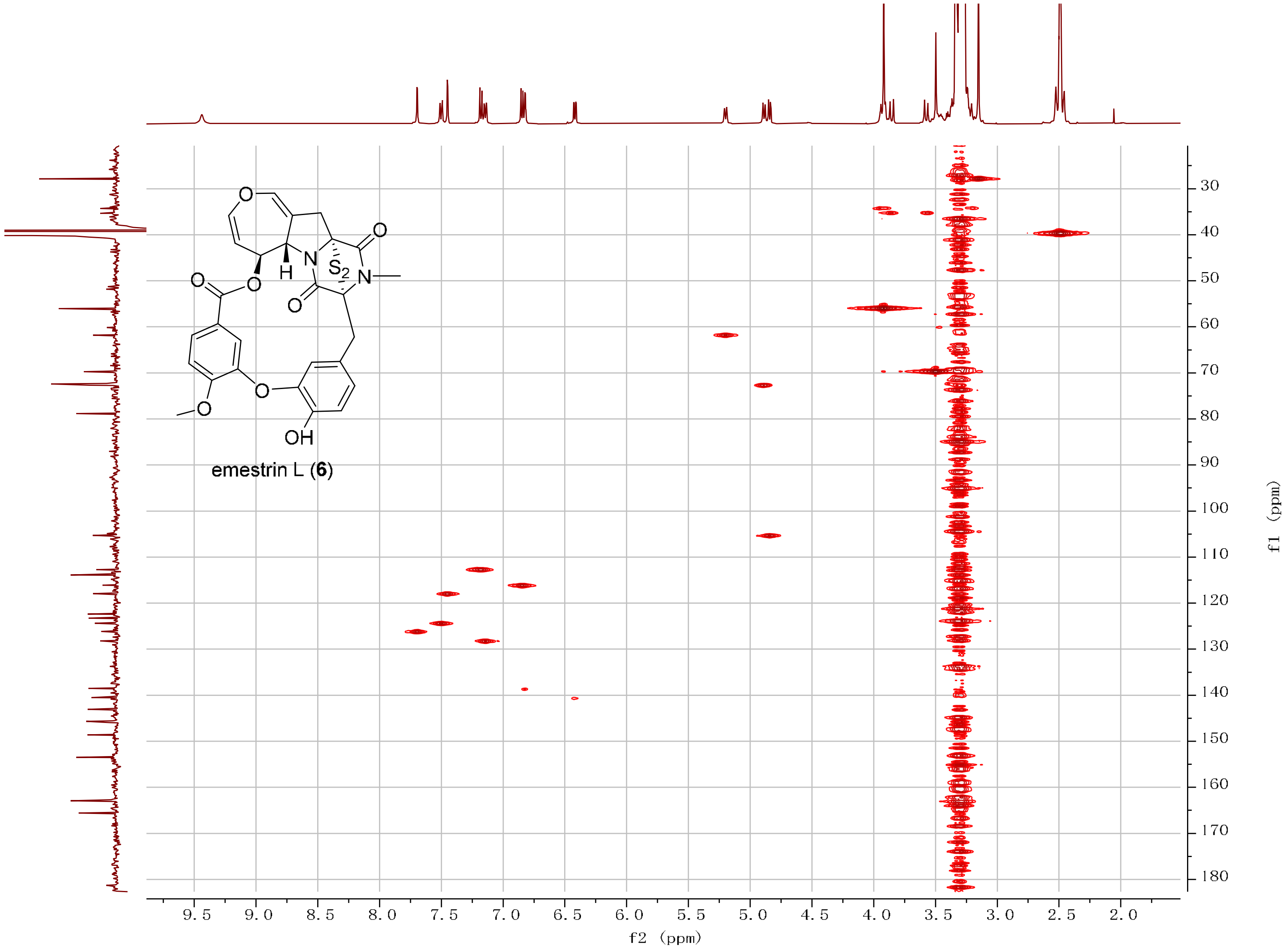

Figure S28. The HSQC spectrum of emestrin L (**6**) in DMSO-d_6.

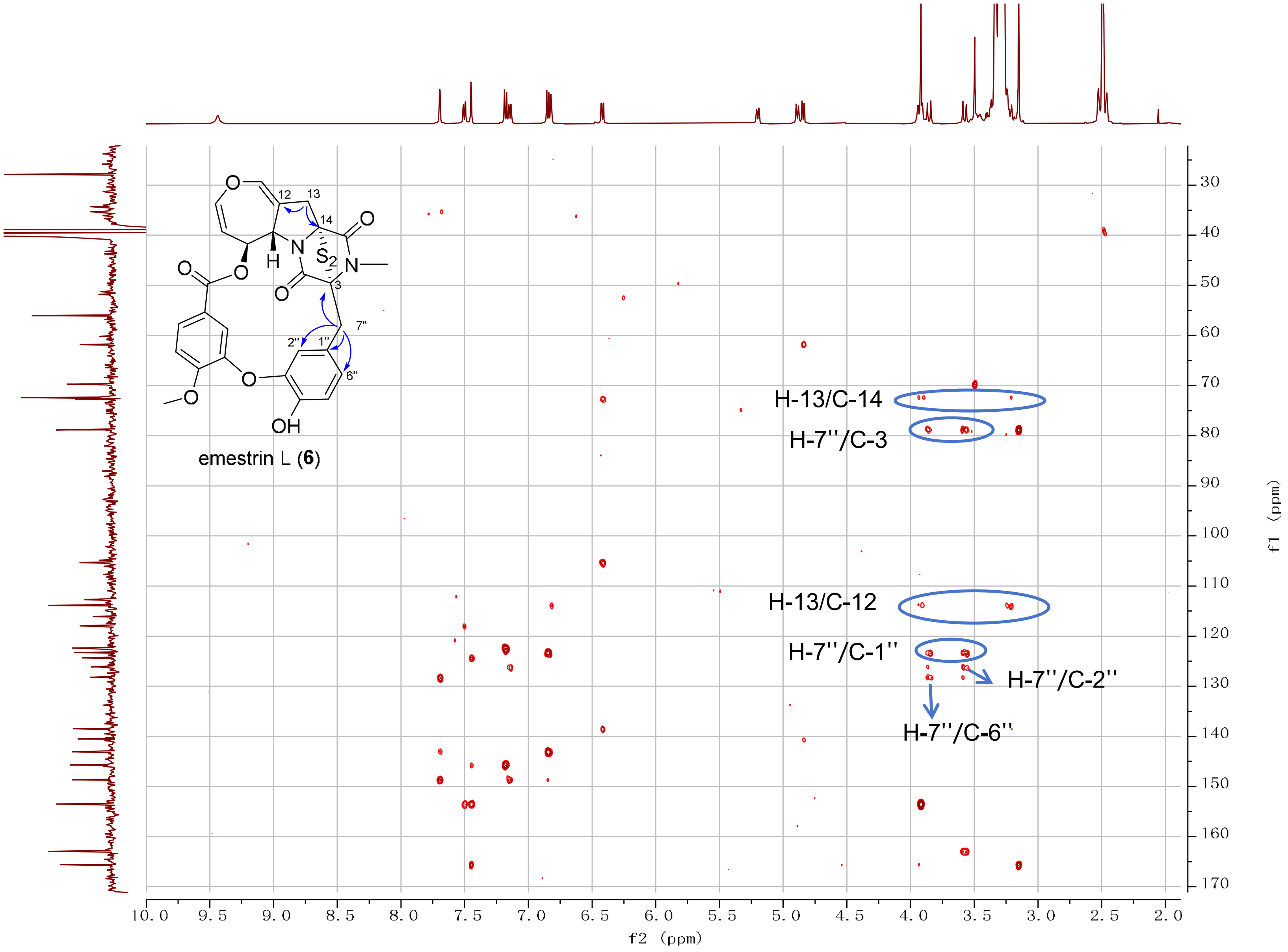

Figure S29. The HMBC spectrum of emestrin L (**6**) in DMSO-d_6.

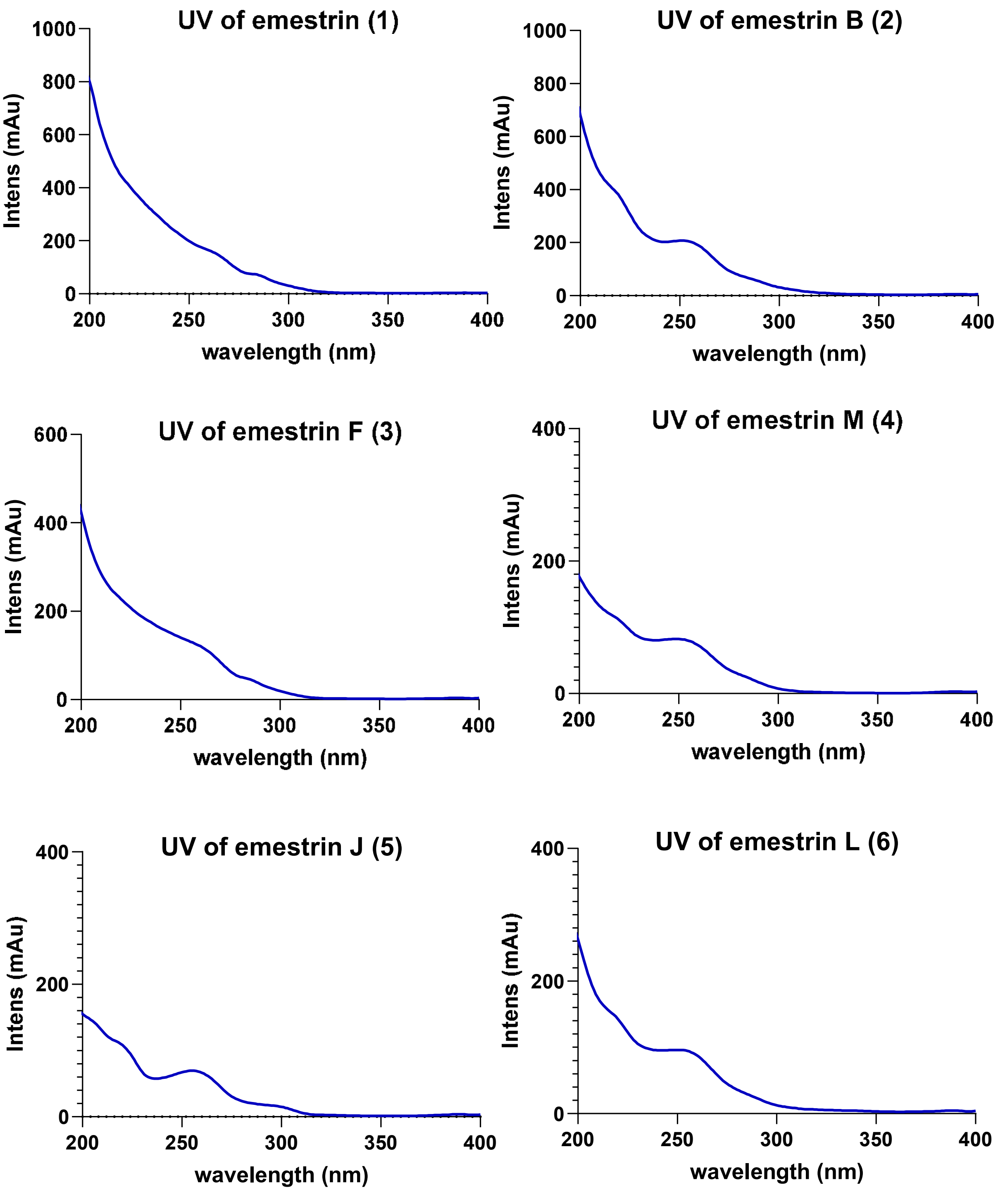

Figure S30. The UV spectra of compounds **1−6** in ACN.

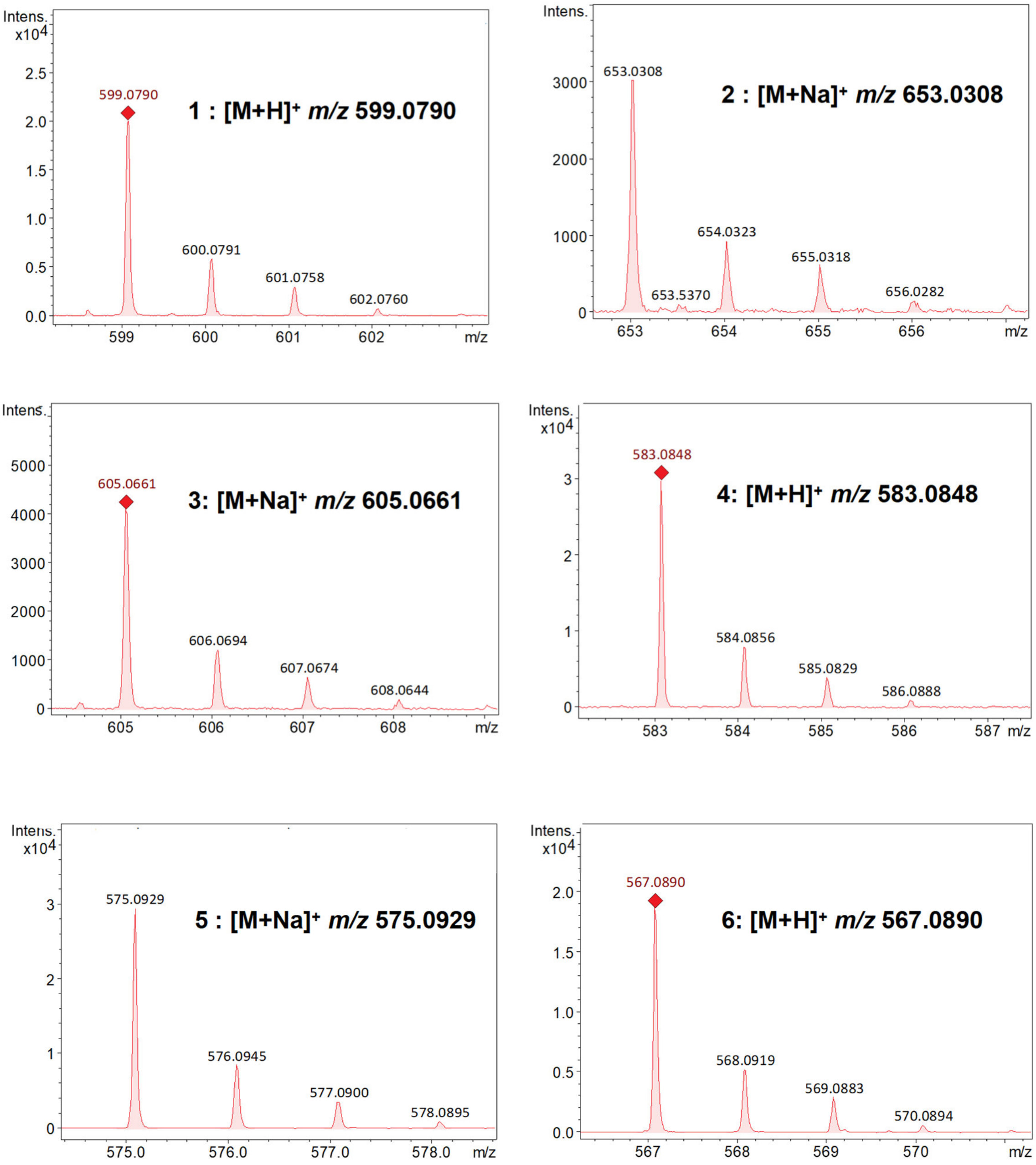

Figure S31. Isotopic pattern of $[M+H]^+$ or $[M+Na]^+$ ions of compounds **1−6**.

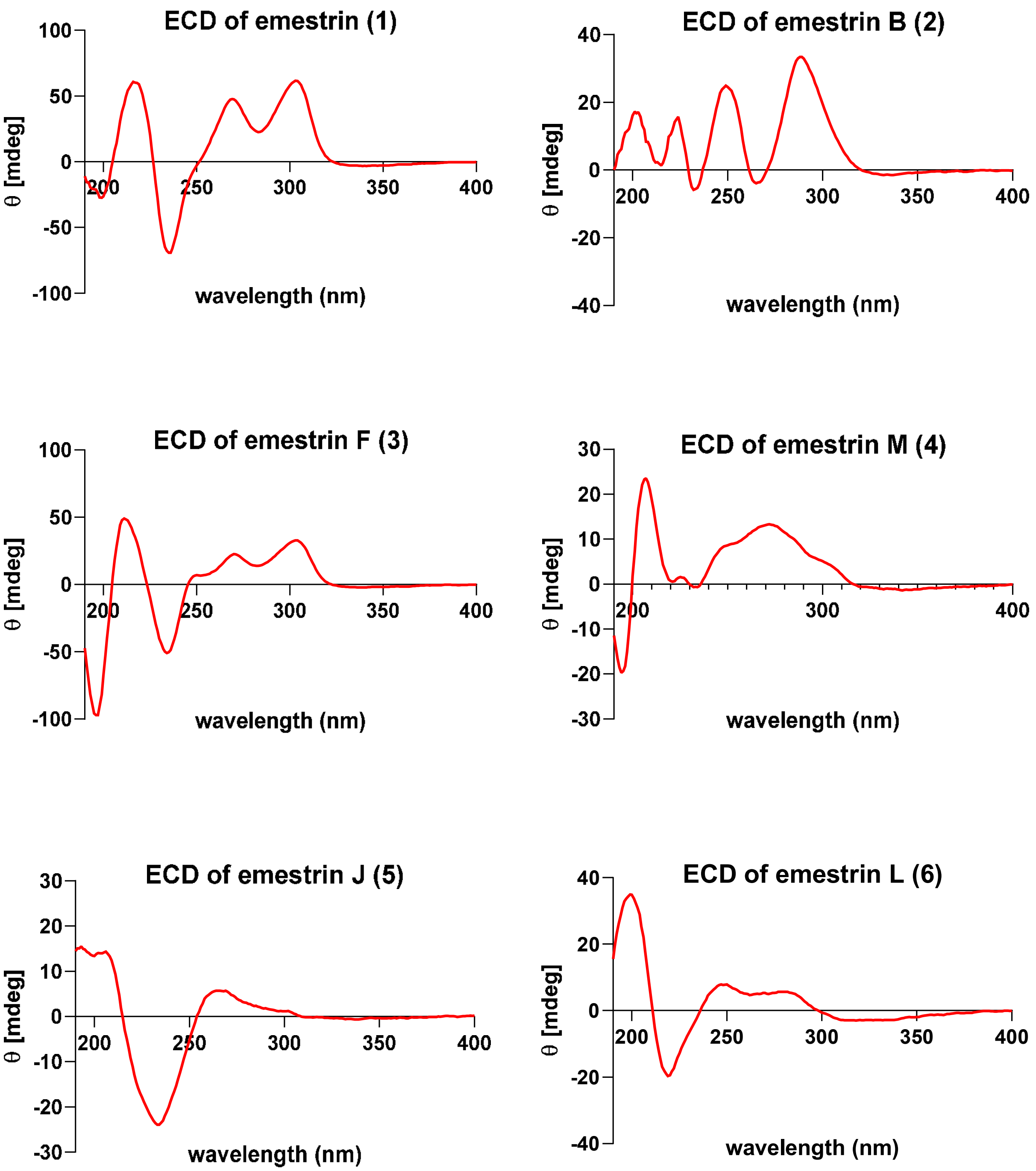

Figure S32. The experimental ECD spectra of compounds **1−6** in ACN.

References

[1] H. J. Lim, E.-H. Lee, Y. Yoon, B. Chua, A. Son, *J. Appl. Microbiol.* **2016**, *120*, 379.
[2] M. R. Green, J. Sambrook, *Molecular cloning: a laboratory manual*, Cold Spring Harbor Laboratory Press, Cold Spring Harbor, New York, **2012**.
[3] J. Fan, G. Liao, F. Kindinger, L. Ludwig-Radtke, W.-B. Yin, S.-M. Li, *J. Am. Chem. Soc.* **2019**, *141*, 4225.
[4] F. Kindinger, J. Nies, A. Becker, T. Zhu, S.-M. Li, *ACS Chem. Biol.* **2019**, *14*, 1227.
[5] S. A. Stierle, S.-M. Li, *J. Fungi.* **2022**, *8*, 493.
[6] O. V. Dolomanov, L. J. Bourhis, R. J. Gildea, J. A. K. Howard, H. Puschmann, *J Appl Crystallogr* **2009**, *42*, 339.
[7] G. M. Sheldrick, *Acta Crystallogr. A* **2008**, *64*, 112.
[8] G. M. Sheldrick, *Acta Crystallogr. C* **2015**, *71*, 3.
[9] S. Chang, M. Cai, T. Xiao, Y. Chen, W. Zhao, L. Yu, R. Shao, W. Jiang, T. Zhang, M. Gan et al., *Org. Lett.* **2022**, *24*, 5941.
[10] Y. Li, Q. Yue, N. M. Krausert, Z. An, J. B. Gloer, G. F. Bills, *J. Nat. Prod.* **2016**, *79*, 2357.
[11] N. Koohei, U. Shun-Ichi, N. Shoichi, K. Ken-Ichi, *Chem. Pharm. Bull (Tokyo).* **1987**, *35*, 3460.
[12] H. M. T. B. Herath, M. Jacob, A. D. Wilson, H. K. Abbas, N. P. D. Nanayakkara, *Nat. Prod. Res.* **2013**, *27*, 1562.
[13] Y.-M. Chiang, C. E. Oakley, M. Ahuja, R. Entwistle, A. Schultz, S.-L. Chang, C. T. Sung, C. C. C. Wang, B. R. Oakley, *J. Am. Chem. Soc.* **2013**, *135*, 7720.
[14] M. Nei, S. Kumar, *Molecular Evolution and Phylogenetics*, Oxford University Press, **2000**.
[15] D. T. Jones, W. R. Taylor, J. M. Thornton, *Bioinformatics.* **1992**, *8*, 275.
[16] S. Kumar, G. Stecher, M. Li, C. Knyaz, K. Tamura, *Mol. Biol. Evol.* **2018**, *35*, 1547.
